# Molecular mechanisms of therapeutic resistance in areca nut–associated oral squamous cell carcinoma: the interplay of chronic inflammation and epithelial–mesenchymal transition

**DOI:** 10.3389/fonc.2026.1906691

**Published:** 2026-08-10

**Authors:** Kaushik Joshi, Arya P. R., Dipali Raju, Shilpa H. D., Vijetha Shenoy Belle, Naveena A. N. Kumar, Mehta Vedant Kamal

**Affiliations:** 1 Department of Biochemistry, Dr. Chandramma Dayananda Sagar Institute of Medical Education and Research, Dayananda Sagar University, Deverakaggalahalli, Karnataka, India; 2 Department of Surgical Oncology, Kasturba Medical College, Manipal Academy of Higher Education, Manipal, Karnataka, India; 3 Department of Biochemistry, Kasturba Medical College, Manipal Academy of Higher Education, Manipal, Karnataka, India

**Keywords:** areca nut, chronic inflammation, epithelial-mesenchymal transition, oral squamous cell carcinoma, treatment resistance

## Abstract

Oral squamous cell carcinoma (OSCC) is a significant form of oral cancer prevalent in South and Southeast Asia, where areca nut chewing is widespread. Chronic exposure to areca nut alkaloids, such as arecoline, contributes to oral carcinogenesis by inducing oxidative stress, DNA damage, chronic inflammation, fibrosis, and epithelial-mesenchymal transition (EMT). These pathological changes facilitate the development of potentially malignant lesions, including oral submucous fibrosis, and promote tumour progression, metastasis, immune evasion, and treatment resistance. Several molecular pathways, including NF-κB, MAPK/ERK, TGF-β, Wnt/β-catenin, and Notch signalling, are integral to areca nut-associated carcinogenesis. Persistent inflammatory signalling and EMT contribute to a tumour promoting microenvironment, characterised by extracellular matrix remodelling, stromal activation, and cancer stemness. Changes associated with EMT, such as the loss of E-cadherin and increased expression of N-cadherin and vimentin, enhance cellular migration and invasion and resistance to treatment. Chronic inflammation and EMT contribute to chemoresistance and radiotherapy resistance through mechanisms such as autophagy induction, hypoxia, anti-apoptotic signalling, and the maintenance of cancer stemness. Recent evidence indicates that targeting inflammatory mediators, reactive oxygen species (ROS), and EMT related pathways may enhance therapeutic outcomes in OSCC associated with areca nut consumption. This review summarises the molecular mechanisms linking areca nut exposure to OSCC progression, focusing on the interaction between chronic inflammation and EMT, and examines their implications for tumour aggressiveness, therapeutic resistance, and future targeted therapeutic strategies.

## 1 Introduction

Oral Squamous cell carcinoma (OSCC) is a major oral cancer observed in South and Southeast Asian countries (1). Oral Cancers form one of the major causes of mortality and morbidity, with associated risk factors such as alcohol consumption, smoking, betel quid (areca nut) chewing, and exposure to UV rays (2). Oral cancer prevalence is highest in South and Southeast Asia, with a prevalence of 35-40% of all malignancies in India, 9% in Taiwan, and 2-4% in the West (3, 4). Areca nut is chewed by almost 600 million individuals worldwide (5) with a prevalence ranging from 0.8% to 46.3% in South Asia and 0.4 – 43.5% in Southeast Asia (6). Areca nut chewing habit is more prevalent in females, and studies have shown a higher risk of OSCC among areca nut chewers than among non-chewers (3).

Areca nut chewing releases several alkaloids, including arecoline. It undergoes nitrosation and generates nitrosamines with oncogenic properties that induce DNA damage. This damage results in the formation of reactive oxygen species (ROS) and proinflammatory cytokines (1). Areca nut extracts can promote epithelial-mesenchymal transition (EMT) by improving tumour cell migration and synthesising matrix metalloproteinases (MMP) via the Mitogen Activated Protein Kinase/Extracellular Signal Regulated Kinase (MEK/ERK) signalling pathway, along with disrupting cellular pathways (7). They also modulate inflammatory signalling via prostaglandin endoperoxide synthase (PTGS) and activate oncogenic signalling through the MEK/ERK pathway (7). There is an upregulation of MMP-8, which promotes extracellular matrix degradation and tumour cell migration, thereby enhancing the malignant potential. Activation of the MEK/ERK and MMP pathways facilitates EMT, further contributing to disease progression (7).

Despite advances in treatment protocols, OSCC continues to show poor outcomes owing to increasing chemoresistance, radiotherapy resistance, and recurrence (3). Chronic areca nut exposure induces stemness and autophagy, reducing sensitivity to agents such as cisplatin and contributing to treatment failure. EMT increases cellular motility by reducing cell adhesion (8). Areca nut chewing promotes the expression of α-smooth muscle actin (α-SMA) and connective tissue growth factor (CTGF), also increases Transforming Growth Factor - β1 (TGF-β1) and promotes cross-linking of collagen fibres, thus reducing susceptibility to collagenase (7, 8). This review aims to understand how areca nut chewing behaviour contributes to treatment resistance in OSCC, focusing on inflammatory and EMT pathways. Chronic areca nut chewing activates pathways such as ROS/AMP Activated Protein Kinase (ROS/AMPK), and MEK/ERK, promoting EMT and cancer stemness (6). These alterations enhance tumour survival and resistance, making these pathways important for understanding and addressing treatment resistance in OSCC.

## 2 Areca nut exposure and use patterns

### 2.1 Chemical composition and bioactive alkaloids

Areca nut extract alkaloids exert genotoxic and cytotoxic effects on human cells. Areca nut extract contains saccharides (26-47%), polyphenols (11-26%), fats (1.3-17%), and alkaloids (0.15-0.67%). Although present in smaller percentages, alkaloids are the most active ingredients associated with pathological development (7). Arecoline is a prevalent and pathological alkaloid. It is converted to arecaidine by salivary enzymes, along with other nitroso compounds that promote 7,12-Dimethylbenz[a]anthracene (DMBA)-induced tumour formation. Other well-known alkaloids include guvacine and guvacoline (1, 2, 7).

For clarity, “areca nut” refers to the seeds of *the Areca catechu*. “Betel quid” refers to a preparation containing areca nut wrapped in betel leaf with slaked lime and may or may not contain tobacco, depending on regional practices. “Gutkha” is a commercial preparation containing areca nut, tobacco, slaked lime, and flavouring agents, whereas paan masala generally contains areca nut without tobacco, although formulations vary geographically. In this review, the term “areca nut-associated” was used only when the cited evidence specifically investigated areca nuts, arecoline, or areca nut extract.

Arecoline induces DNA damage and contributes to the downregulation of p53, which plays a key role in the tumourigenesis of areca nut-associated malignancies (2). It plays a key role in inducing DNA strand breaks, sister chromatid exchanges, micronucleus formation, and chromosomal aberrations (2, 6).

### 2.2 Chewing practices and population patterns

Areca nut is used by approximately 600 million people globally, predominantly from South and Southeast Asian countries, with approximately 20-40% of users from India, Nepal, and Pakistan (2). Areca nuts can be chewed raw or processed by roasting, drying, soaking, or boiling (2). In India, different varieties of preparations are used, including Neetadaka, Chali, Gutkha, Parcha, Kalipak, Nuli, Supari, Tamul, and Bura Tamul (9). The most common form of areca nut chewing is a combination of areca nut, betel leaf, and slaked lime (2). It is also used as an aphrodisiac and appetite suppressant, and in the treatment of cough, fainting, glaucoma, impotence, and leprosy (10).

Areca nut chewing is more common among women than among men. It is commonly used to relieve stress and has addictive properties (10). The use of areca nuts is predominantly seen above the age of 15 years in South and Southeast Asian countries (9). Areca nuts are psychoactive substances that can lead to addiction and dependence in chronic users. Self-employed individuals and housewives are at a higher risk of developing OSCC (9, 10). In India, dry tobacco areca nut preparations include paan masala, gutkha, and mawa. When areca nut is chewed without tobacco, it acts as the main psychoactive substance (5).

## 3 Areca nut driven OSCC pathogenesis

### 3.1 Molecular and genetic alterations

Chronic areca nut exposure promotes stromal remodelling through fibroblast activation, increased α-SMA and CTGF expression, and excessive collagen deposition (11). Arecoline and its metabolites further alter epithelial stromal interactions by enhancing inflammatory and profibrotic signalling (11). These early microenvironmental changes establish a biological context for chronic inflammation, epithelial plasticity, and subsequent malignant progression (12).

Genetic changes play a crucial role in arecoline induced carcinogenesis. This occurs via DNA damage, altered gene expression, oncogenic activation, and promotion of cancer stemness (11, 12). Experimental studies have demonstrated increased levels of markers of DNA double-strand breaks, such as γH2AX and p53 binding protein-1 (53BP1), indicating direct genomic injury (13). Areca nut extracts induce the dysregulation of tumour suppressor genes, including TP53, Dual specificity phosphatase 4 (DUSP4), and retinoic acid receptor beta (RARB), promoting loss of growth control and unchecked proliferation (1). Upregulation of proto-oncogenes such as Myelocytomatosis viral oncogene homolog (MYC), Neurogenic locus notch homolog protein-1 (NOTCH1), Cysteine rich angiogenic inducer 61 (CYR61), and Discoidin domain receptor-1 (DDR1) promotes sustained proliferative signalling and tumour progression, leading to genomic instability (1). Genetic susceptibility to polymorphisms in genes such as collagen type 1 alpha 1/alpha2 (COL1A1, COL1A2), transforming growth factor beta-2 (TGF-β2), vascular endothelial growth factor (VEGF), and hypoxia inducible factor 1- alpha (HIF-1α) modulates the risk of developing oral cancer (12). These genetic variations promote fibrosis, angiogenesis, and the tumour microenvironment (TME), thereby increasing the risk of oral cancer development (14).

Epigenetic mechanisms involve extensive gene expression reprogramming via microRNAs (miRNAs), DNA methylation, and transcriptional regulation (13). Arecoline alters miRNA profiles by upregulating oncogenic miRNAs, such as 23a, miR-10b, and miR-886-3p, and downregulating tumour suppressive miRNAs, such as miR-145, the miR-200 family, and miR-22 (12). DNA methylation alters the methylation of PTK6, influencing carcinogenic pathways. Arecoline also modulates the transcription factors involved in EMT pathways, including ZEB1, Snail, and Twist (15). This promotes invasion and metastasis of the tumour. The key pathways affected include Epidermal Growth Factor Receptor/Phosphoinositide 3-kinase/Protein kinase B (EGFR/PI3K/Akt), Mitogen Activated Protein kinase (MAPK), Wingless related integration site (WNT), and NOTCH1, which regulate the proliferation and survival of cancer cells (15).

These epigenetic modifications not only alter individual gene expression but also collectively reprogramme oral epithelial cells towards a pro-tumourigenic phenotype (12). The downregulation of tumour-suppressive miRNAs, notably the miR-200 family and miR-145, alleviates the inhibition of EMT-associated transcription factors and stemness regulators, thereby facilitating epithelial plasticity, invasion, and the acquisition of cancer stem cell (CSC) characteristics (12, 13). In contrast, the overexpression of oncogenic miRNAs, such as miR-23a and miR-10b, augments cellular proliferation, migration, and resistance to apoptosis (15). Additionally, aberrant DNA methylation contributes to malignant transformation by modifying the transcriptional regulation of tumour suppressor genes and enabling sustained activation of the EGFR/PI3K/Akt, MAPK, Wnt/β-catenin, and NOTCH signalling pathways (15). Collectively, these epigenetic alterations create a permissive molecular environment that fosters the progression from oral potentially malignant disorders (OMPD) to invasive OSCC, while also contributing to therapeutic resistance through the maintenance of EMT and cancer stemness (15).

### 3.2 Tumour microenvironment remodelling

The TME in areca nut-associated carcinogenesis involves interactions between epithelial cells, stromal fibroblasts, and immune mediators (13). Arecoline exposure to keratinocytes and fibroblasts in the oral mucosa increases the levels of fibrosis associated proteins such as TGF-β1, fibronectin, and collagen, indicating stromal fibroblast activation and extracellular matrix remodelling, along with an increase in the expression of MMP9, which contributes to tissue invasion and degradation of the extracellular matrix (13). Experimental investigations have revealed that exposure to areca nuts is linked to the differential expression of extracellular matrix components. Notably, there is an upregulation of COL1A1, COL1A2, collagen type III, fibronectin, and α-smooth muscle actin, which is accompanied by the activation of CTGF and TGF-β signalling pathways (13). In addition to excessive collagen synthesis, arecoline facilitates collagen crosslinking and reduces collagen degradation (13). This leads to the progressive accumulation of the extracellular matrix and an increase in tissue stiffness (13). These alterations contribute to fibroblast activation, myofibroblast differentiation, and the development of a fibrotic TME characteristic of oral submucous fibrosis (OSF) (12). Cytokines such as Interleukin -1β (IL-1β), Interleukin 6 (IL-6), Interleukin - 8 (IL-8), and Interleukin -17 (IL-17), and chemokines such as C-C Motif Chemokine Ligand 2 (CCL2) and C-C Motif Chemokine Ligand 5 (CCL5) promote an inflammatory microenvironment by promoting tumour progression and immune remodelling (12, 13). Signalling pathways, such as the nuclear factor kappa light chain enhancer of activated B (NF-κB) and MAPK, are activated to sustain inflammation and cell survival (1). Areca nut extracts also promote ROS generation and enhance oxidative stress in cells, thereby supporting a tumour-promoting microenvironment (1). Thus, the TME is characterised by fibrosis, stromal activation, chronic inflammation, and signalling dysregulation, contributing to the development of oral cancer.

### 3.3 Field cancerisation and multifocal risk

Field cancerisation is associated with the presence of genetically altered, but clinically normal mucosa around the tumour (15). These altered fields may persist even after surgical excision of the tumour, while maintaining an adequate surgical margin of clearance, thus increasing the risk of tumour recurrence (15). Field cancerisation occurs through the clonal expansion of a single genetically altered CSC that proliferates and produces unstable daughter cells, gradually replacing the normal epithelium (16).

Field cancerisation explains the development of premalignant and malignant lesions in the oral mucosa due to widespread carcinogenic exposure. Diffuse molecular alterations are observed when the oral mucosa is chronically exposed to areca nut-induced carcinogens, such as arecoline, ROS, and nitrosamines (2). The oral mucosa is continuously exposed to these carcinogens when betel quid is retained in the mouth for a long time, thus promoting epithelial injury and contributing to the formation of multiple OPMDs (2). These potentially malignant lesions can occur simultaneously at different sites on the oral mucosa. Arecoline induces genotoxicity, DNA damage, and epithelial changes, contributing to transformation across various mucosal fields of the oral cavity. ROS-induced DNA injury, altered transcription, and activation of oncogenic pathways also play key roles in the molecular changes in oral mucosa (12). These triggers expose multiple mucosal areas to malignant transformations. Field cancerisation in oral cancer is mainly due to the exposure of the mucosa to carcinogens, which causes diffuse epithelial injury and genetic and molecular alterations (16).

Multiple molecular markers have been implicated in field cancerisation, including p53 mutations, Ki-67 overexpression, chromosomal instability, and heterozygosity loss. The identification of these markers in premalignant lesions of the oral cavity helps identify high risk groups that may undergo malignant transformation (16). Histopathological studies have demonstrated that clinically normal mucosa near OSCC exhibits epithelial dysplasia, hyperkeratosis, and premalignant changes (15). Field cancerisation also explains the multifocal nature of oral potentially malignant lesions and oral cancers, where multiple independent lesions can arise at various sites simultaneously within the carcinogen exposed mucosal field (16).

## 4 Areca nut induced inflammatory signalling

### 4.1 Chronic inflammation and tissue injury

Areca nut chewing results in a combination of mechanical injury, chemical toxicity and immune activation in the oral cavity (16). Over time, this leads to persistent mucosal damage, chronic inflammation, fibrosis, and a significant increase in the risk of oral cancer (17). The abrasive fibres of betel nuts physically damage the oral epithelium, causing chronic inflammation and disruption of the epithelial barrier (18). Areca alkaloids and phenols induce apoptosis in oral mucosal cells, oxidative stress, DNA damage, and epithelial atrophy, predisposing individuals to lesions and carcinogenesis (7). Arecoline and other components of areca promote the transition of fibroblasts to myofibroblasts, excessive collagen deposition, and submucosal scarring, known as OSF (11).

Areca nut extract induces the expression of COX-2, PGE_2_, IL-6, TNF-α, IL-8, and other cytokines in oral epithelial cells, thereby promoting juxta-epithelial inflammation characteristic of oral OSF and leucoplakia (7). Aqueous extracts of areca mobilise intracellular Ca^2+^ in various immune cell lines, leading to proliferation and the release of pro-inflammatory cytokines, which contribute to the maintenance of a chronic inflammatory microenvironment in the oral cavity (19). Areca nut components impair T-cell activation and neutrophil function, alter lymphocyte subsets, and diminish cell mediated immunity (CMI), facilitating the persistence of lesions and tumour immune evasion (20). Specific epithelial subsets with pro-inflammatory and pro-fibrotic profiles interact with T cells, for instance, through the MIF–CD74/CXCR4 pathway, promoting fibrosis (21). Dental pulp stem cells have the potential to partially restore immune homeostasis in experimental models (22). Repeated mastication results in continuous tissue insult, thereby impeding normal healing processes and perpetuating a cycle of inflammation, fibrosis, and atrophy, facilitating the penetration of areca toxins (23). Additionally, areca nut chewing modifies the oral microbiota, with an increase in Streptococcus species associated with mucosal injury and OSF in murine models, promoting chronic periodontal inflammation and periodontitis (24). Clinically, these factors contribute to keratinisation of the mucosa, OSF, leucoplakia, and a significantly elevated risk of oral cancer, with a dose-response relationship observed concerning the duration and intensity of chewing (25).

### 4.2 Core inflammatory pathways

Areca nut and its alkaloid, arecoline, are implicated in driving chronic inflammation and DNA damage within the oral mucosa, thereby facilitating the progression from precancerous lesions to OSCC (26). Three principal inflammatory pathways have been consistently identified are NF-κB signalling, COX-2/prostaglandins, and pro-inflammatory cytokines, including IL-6, TNF-α, and IL-1β (27). Areca nut extract has been shown to activate NF-κB in oral keratinocyte lines, characterised by biphasic NF-κB activity, IκBα degradation, and nuclear accumulation of NF-κB (28). The biphasic pattern of NF-κB activation consists of an initial transient phase, which is characterised by the degradation of IκBα and nuclear translocation of NF-κB (28). This phase leads to the induction of inflammatory mediators, including COX-2, IL-6, and TNF-α. Subsequently, a sustained phase occurs, which is dependent on ROS and MAPK/ERK pathways (7). This phase perpetuates NF-κB activation through cytokine feedback, thereby facilitating chronic inflammation, fibrosis, EMT, and malignant progression (7). Inhibition of NF-κB resulted in the suppression of areca-induced COX-2 upregulation, thereby establishing a direct link between NF-κB and downstream inflammatory enzymes in these cells (7). ROS generated by areca components can activate the IκB/NF-κB pathway via MAPK/ERK, thereby sustaining the transcription of pro-inflammatory genes and inducing keratin changes that impede wound healing and promote cancer progression (29). NF-κB-regulated mRNA or lncRNA networks, enriched for immune and inflammatory pathways, are prominent in areca-associated tongue squamous cell carcinoma, underscoring the role of inflammation-driven carcinogenesis (30).

**Figure 1:**
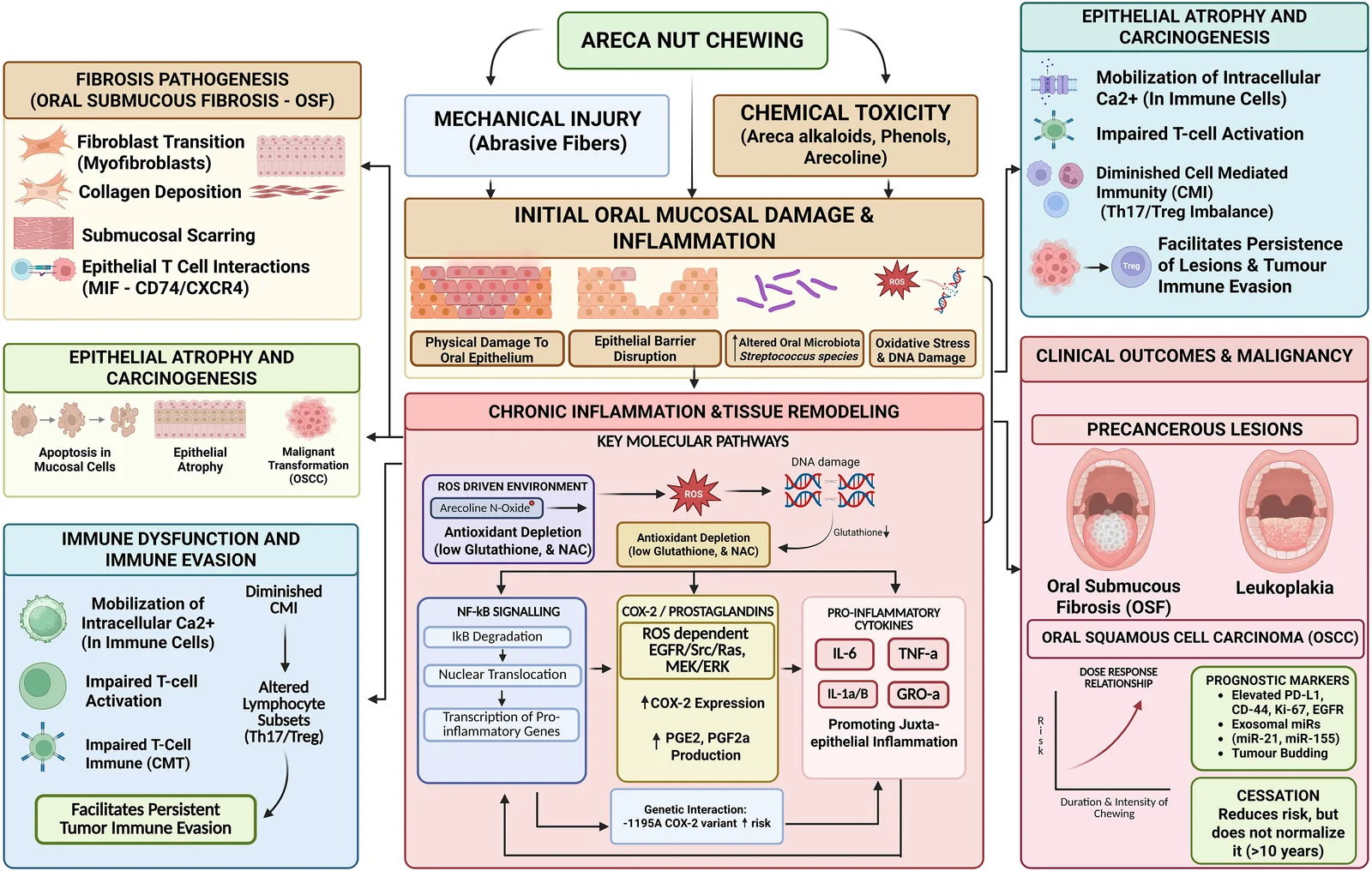
The pathogenesis of oral carcinogenesis induced by areca nut involves both mechanical injury and chemical toxicity, as illustrated in the accompanying image. Areca nut chewing initiates a cycle that leads to chronic inflammation, oxidative stress, and tissue remodelling. These processes contribute to malignant transformation primarily through the activation of the NFkB pathway, while simultaneously promoting immune evasion by impairing T-cell activation. The progression from precancerous lesions to aggressive OSCC demonstrates a relationship dependent on exposure, which is further aggravated by persistent immune dysfunction and alterations in the microenvironment.

Areca nut extract and arecoline strongly induce COX-2 expression and PGE_2_/PGF_2_α production in oral keratinocytes through ROS-dependent EGFR/Src/Ras and MEK/ERK signalling (31). Genetically, a COX-2 promoter variant (-1195A) interacts with betel chewing to greatly increase OSCC risk, consistent with COX-2 being a key areca-responsive inflammatory driver (32). IL-6 which is strongly induced by areca nut extract in keratinocytes and fibroblasts, promoting EMT, cell proliferation, and malignant transformation; antioxidants or IL-6 neutralisation reduce EMT markers (33). Areca induced TNF-α and interleukin 1α/interleukin 1β (IL-1α/IL-1β) expression, and IL-1 can drive TNF-α and IL-6, while IL-1β and other cytokines are elevated in OSCC tissues of areca users and in ANO-treated models (34). Conditioned media derived from fibroblasts exposed to areca, rich in IL-6, IL-8, and GRO-α, induce ROS dependent DNA damage in keratinocytes, establishing a connection between the stromal microenvironment and epithelial mutagenesis (35–37). Collectively, NF-κB activation, COX-2/prostaglandins, and a cytokine-rich, ROS-driven environment contribute to chronic inflammation, facilitating OSF, immune evasion, and ultimately, OSCC. Major pathways associated with inflammation-driven carcinogenic pathways induced by areca nut chewing behaviour are illustrated in **Figure 1**.

### 4.3 Interaction with areca nut components

Arecoline, the principal alkaloid of areca nut, significantly disrupts the redox equilibrium in oral cells and promotes an inflammatory cytokine environment (25). This ROS cytokine axis links betel chewing to compromised healing, fibrosis, and oral cancer (26). In normal gingival keratinocytes and S-G epithelial cells, low to moderate concentrations of arecoline induced mitochondrial ROS, which subsequently activated the MAPK/ERK and NF-κB signalling pathways (38). Arecoline and its metabolite, ANO, also elevate ROS levels and deplete antioxidants, such as glutathione and NAC, in OSCC cells and fibroblasts (13). Downstream, ROS modulates cell cycle proteins (p21 and p27 via ROS/mTORC1) and DNA damage, which facilitates malignant progression. Arecoline increased the expression of IL-1α/β, IL-6, IL-8, IL-17, TNF-α, and TGF-β1 in keratinocytes, fibroblasts, and OSCC cells, often through ROS-dependent NF-κB and MAPK activation (39).

In S-G epithelial cells, exposure to 12.5 µg/mL arecoline results in the induction of ROS and the transcription of TNF-α and IL-6 (29, 40). ROS, MAPK/ERK, and NF-κB pathways facilitate the expression of KRT6, IL-6, and TNF-α, thereby impairing cellular migration and wound healing processes (41). Fibroblasts exposed to areca nut secrete GRO-α, IL-6, and IL-8, which in turn cause ROS-mediated DNA damage in adjacent keratinocytes through NADPH Oxidase 1 (NOX1)/4 activation (41). Arecoline notably upregulates IL-1β, IL-6, IL-8, IL-17a, CCL2, G-CSF, and NF-κB in OSCC and fibroblasts, establishing a connection between ROS, EMT, and fibrosis (42). The interplay between ROS and cytokine networks underpins chronic inflammation, fibrosis, immune evasion, and metastasis.

### 4.4 Clinical consequences and risk markers

Areca nut chewing is a recognised etiological factor in the development of OMPDs, notably OSF and leucoplakia, which often precede OSCC. Chronic exposure to areca nut alkaloids and related carcinogens induces epithelial dysplasia, fibrosis, persistent inflammation, and molecular changes that promote malignant transformation of the oral epithelium (3). OSCC frequently arises from OSF and leucoplakia, both of which pose a significant risk of malignant transformation, contributing to an earlier onset and considerable disease burden in endemic regions (43). Histological characteristics, including tumour size, mode of invasion, and the most aggressive invasion pattern, are strong predictors of locoregional metastasis (4, 44–46).

Immune and molecular markers, such as elevated Programmed Death Ligand-1 (PD-L1) expression, T helper cell (Th17)/Regulatory T cell (Treg) imbalance, EGFR overexpression, CD44, and Ki67, are consistently associated with lymph node metastasis, recurrence, and reduced survival rates (47, 48). Additionally, exosomal miRNAs, including miR-21 and miR-155, as well as Arginase-1, are associated with metastasis and poor prognosis, indicating a pro-invasive and immunosuppressive TME (49). Although discontinuing areca nut chewing significantly lowers the risk of oral cancer, the risk remains higher than that of individuals who have never chewed, even after extended abstinence (50). Those who have ceased chewing for over ten years exhibit a lower risk than current chewers, underscoring the significance of areca nut cessation as a crucial tertiary prevention strategy following OSCC treatment to reduce disease recurrence and enhance long-term outcomes (51).

## 5 Epithelial–mesenchymal transition in OSCC

### 5.1 Epithelial mesenchymal transition hallmarks and biomarkers

**Figure 2:**
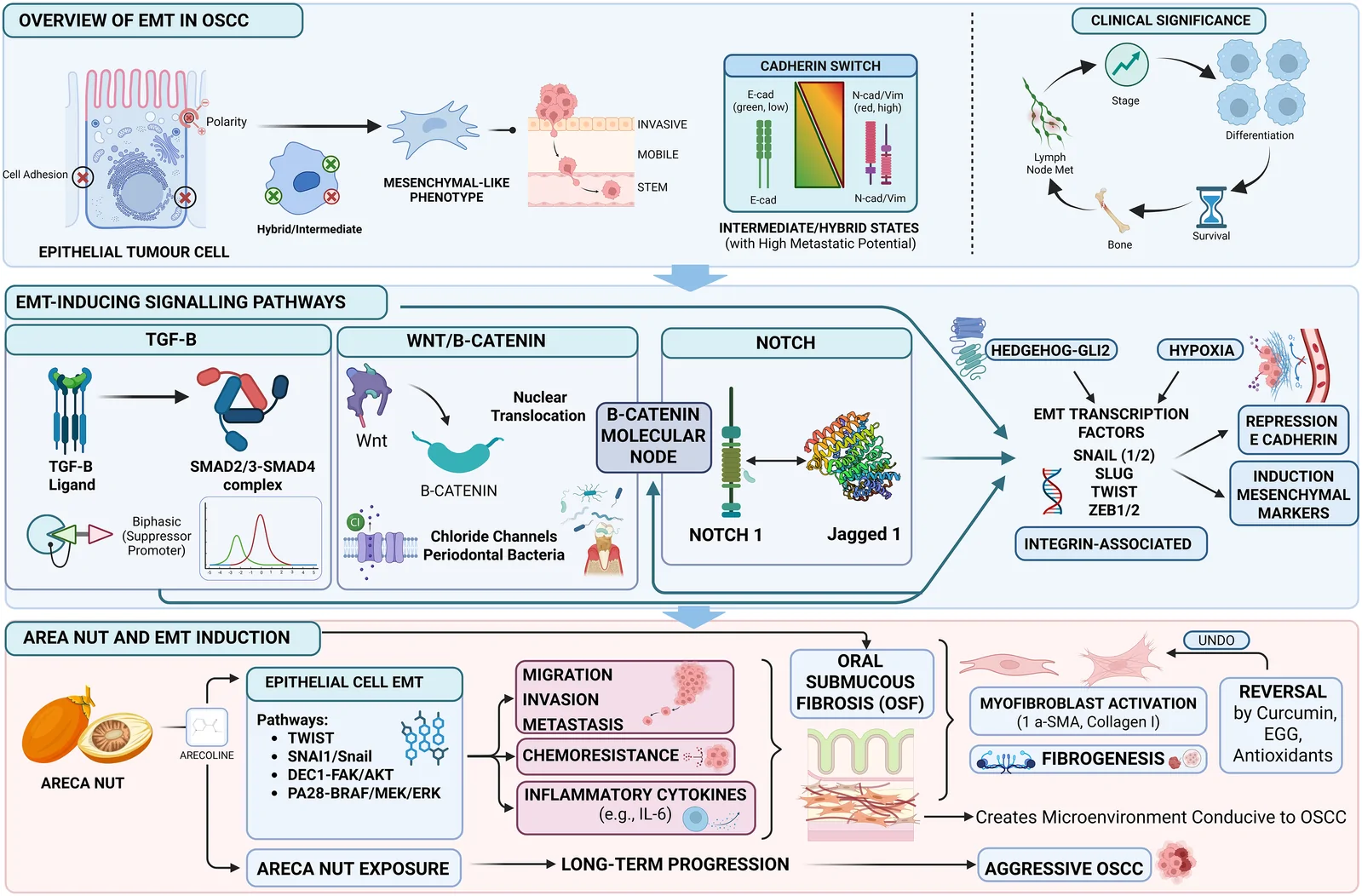
This image depicts the progression of OSCC, initiated by the transition of epithelial cells to a mesenchymal-like phenotype. This transition is driven by key signalling pathways, including TGF-β, WNT/β-catenin, and NOTCH, alongside the activation of EMT transcription factors such as SNAIL and TWIST. Notably, the areca nut and its alkaloid, arecoline, serve as primary catalysts in this process by activating specific molecular pathways. These pathways promote critical cancer hallmarks, including migration, invasion, and chemoresistance (insert reference). Consequently, these alterations establish a microenvironment conducive to the long-term progression toward aggressive OSCC.

EMT in OSCC involves the loss of adhesion and polarity by epithelial tumour cells and the acquisition of a more mobile, invasive, and mesenchymal like phenotype (**Figure 2**) (52). A central feature is the loss or mislocalisation of the epithelial marker E-cadherin with the gain of mesenchymal markers, such as vimentin and N-cadherin, often termed a cadherin switch (52, 53). Reduced E-cadherin expression is common in OSCC compared to normal mucosa and dysplasia and increases with tumour grade or invasiveness (54). It is often accompanied by a shift from membranous to cytoplasmic localisation, reflecting the loss of stable cell–cell adhesion (55). Loss of E-cadherin is repeatedly linked to invasion, lymph node metastasis, bone invasion, and poor survival (56). Vimentin is frequently neo-expressed in dysplasia and OSCC epithelium, often increasing with disease severity and at invasive fronts, indicating mesenchymal phenotypic change (57). N-cadherin expression is elevated in OSCC compared to normal epithelium and tends to increase with poorer differentiation and progression; this E- to N-cadherin shift is a hallmark of EMT in OSCC (58).

Elevated vimentin levels and diminished E-cadherin expression were significantly correlated with lymph node metastasis, advanced disease stage, poor differentiation, bone invasion, and decreased survival (59, 60). The combined assessment of E-cadherin and vimentin levels provides a more accurate prediction of outcomes than that of either marker alone (61). N-cadherin overexpression and cadherin switching were associated with advanced disease stage, nodal positivity, and aggressive invasion patterns (62). N-cadherin and SNAIL2 were overexpressed in node-positive tumours. Certain tumours exhibited co-expression of E-cadherin with either vimentin or N-cadherin at the invasive front, indicating intermediate states with high metastatic potential (63).

### 5.2 EMT signalling pathways

Invasion and metastasis in OSCC are driven by EMT. Three principal and closely interconnected pathways namely TGF-β, Wnt/β-catenin, and Notch are recurrent inducers of EMT in OSCC and other cancers (64). TGF-β signalling is recognised as the most prevalent EMT-inducing pathway, operating through Smad2/3–Smad4 complexes to regulate EMT related genes (65). In murine OSCC, stimulation by TGF-β initiates EMT, enhances cell motility, and upregulates genes associated with EMT and angiogenesis, and its inhibition diminishes tumour angiogenesis *in vivo* (66). In various cancers, TGF-β1 transitions from a tumour-suppressive role (growth arrest and apoptosis) in the early stages to promoting EMT, immune evasion, and metastasis in the later stages (67). Additionally, TGF-β modulates other EMT pathways by forming complexes with β-catenin/Lymphoid Enhancer Binding Factor 1 (LEF1) and activating the Notch ligand Jagged1, thereby linking it to Wnt and Notch signalling (68).

In OSCC, EMT can be driven by Wnt/β-catenin pathway activation (69). Wnt ligands inhibit glycogen synthase kinase-3 beta (GSK-3β), stabilize β-catenin, promote its translocation to the nucleus, and induce the expression of EMT-related genes (67, 69). Blockade of chloride channels in OSCC cells resulted in a mesenchymal morphology, downregulation of E-cadherin, upregulation of vimentin, ZEB1, and Snail, and activation of Wnt/β-catenin signalling, but did not affect TGF-β/Smad signalling. This suggests a Wnt-driven EMT pathway that is independent of TGF-β (70). Periodontal pathogens can also induce EMT in OSCC, characterised by increased expression of TWIST, ZEB1, and N-cadherin, and decreased E-cadherin levels (70). The flavonoid fisetin can partially reverse these changes by reducing β-catenin levels in the canonical Wnt pathway (71). The Wnt/β-catenin pathway is a central regulator of EMT in cancer, coordinating cytoskeletal remodelling, cell motility, and metastasis. β-catenin serves as a molecular node linking Wnt and other EMT pathways, including TGF-β (72). EMT markers, such as the loss of E-cadherin, relocalisation of β-catenin, and increased levels of N-cadherin, vimentin, and TWIST, are closely associated with Wnt activity and are utilized as biomarkers for the progression of oral lesions (73).

Notch signalling, along with TGF-β and Wnt, is a trigger of EMT in cancer and OSCC (68). In OSCC cell lines, specifically Tca8113 and CAL27, Notch signalling activation results in Snail and vimentin upregulation, E-cadherin downregulation, and enhanced cellular migration (74). Inhibition of γ-secretase using DAPT reversed EMT marker expression and diminished migration, suggesting a Notch-dependent EMT-driven metastasis (75). Notch signalling is activated downstream of TGF-β, where TGF-β/Smad complexes can activate Jagged1 and release the Notch intracellular domain, thereby co-regulating EMT gene programmes with TGF-β (76). Single-cell RNA sequencing of TGF-β1 treated epithelial cells revealed a delayed yet robust induction of Notch, Wnt, and other pathways during EMT, with Notch serving as a pivotal driver of TGF-β–induced EMT and hybrid EMT states, which are associated with adverse outcomes (77).

EMT is not driven by a singular pathway; instead, it is the interaction of multiple pathways that play the role in activating and sustaining the transition (78). TGF-β, Wnt/β-catenin, and Notch pathways induce EMT transcription factors like Snail, Slug, TWIST, ZEB1/2 and collectively contribute to the repression of E-cadherin and the induction of mesenchymal markers (79). TGF-β signalling can directly interact with β-catenin and LEF1/TCF and co-regulate extensive EMT gene sets with Notch, demonstrating the profound integration of these pathways (68, 76). In OSCC, additional pathways, such as Hedgehog-GLI2 signalling, hypoxia, and integrin-associated pathways, converge with Wnt/β-catenin and EMT, further enhancing invasive and metastatic behaviour (80).

### 5.3 Areca nut driven EMT

The areca nut and its principal alkaloid, arecoline, induce EMT in oral epithelial cells. Concurrently, they promote fibroblast activation and myofibroblast differentiation (81). This establishes a link between chronic exposure to these substances, OSF, and the subsequent development of aggressive OSCC (81). Although epithelial EMT and fibroblast activation are distinct biological processes, both contribute to collagen-rich fibrosis and collectively create a microenvironment conducive to malignant transformation (82). Arecoline induces EMT in OSCC cell lines, resulting in increased migration, invasion, and cervical lymph node metastasis, accompanied by alterations in inflammatory cytokines and EMT markers (11, 17). Chronic exposure of oral epithelial cells to arecoline leads to the acquisition of EMT characteristics, enhanced migratory and invasive capabilities, stemness, and chemoresistance (83). Mechanistically, arecoline facilitates EMT through multiple pathways, including TWIST, SNAI1/Snail-, DEC1–FAK/AKT-, and PA28γ–BRAF/MEK1/ERK-dependent signalling, characterised by the loss of E-cadherin and gain of N-cadherin and vimentin (11, 13, 82). Similarly, areca nut extract induces EMT-like morphology, upregulation of Snail, production of IL-6, and loss of E-cadherin in oral keratinocytes, which can be reversed by neutralising IL-6 or adding antioxidants (3, 11, 13).

Arecoline promoted fibroblast activation and myofibroblast differentiation in OSF fibroblasts, characterised by increased α-SMA expression, enhanced migration, and upregulation of profibrotic regulators including Snail and Twist (83). Curcumin and Epigallocatechin Gallate (EGCG) reversed the expression of EMT-associated transcription factors, including ZEB1, Twist, and Slug, thereby attenuating areca-associated fibroblast activation and myofibroblast differentiation (83). miR-203 and its targets regulate arecoline induced EMT alterations, characterised by an increase in N-cadherin and vimentin levels and a decrease in E-cadherin expression in keratinocytes (84–86). OSF is a fibrotic premalignant condition primarily induced by areca nut consumption. EMT, together with fibroblast activation and myofibroblast differentiation, plays a role in fibrogenesis and the subsequent development of epithelial dysplasia, which has the potential to progress to malignancy (81, 87). Arecoline, a compound found in areca nuts, is known to facilitate myofibroblast differentiation by upregulating α-SMA and collagen I expression through EMT regulators such as ZEB1, Slug, Snail, and IL-6 positive feedback (17, 88, 89). Persistent activation of fibroblasts and collagen gel contraction mimic the fibrosis characteristics of OSF (17, 81). The prevailing literature indicates that areca nut-induced epithelial EMT, along with fibroblast activation and myofibroblast differentiation, contributes to oral fibrosis, microenvironmental remodelling, and the eventual development of OSCC (90).

Recent experimental evidence suggests that fibrosis plays an active role in malignant transformation, rather than being merely a secondary effect of chronic areca nut exposure (91). The excessive deposition of extracellular matrix results in increased tissue stiffness, which activates mechanotransduction pathways that promote EMT, tumour cell invasion, and cancer stemness (91). Furthermore, the dense, collagen-rich stroma impedes immune-cell infiltration and reduces the penetration of chemotherapeutic agents, while also promoting hypoxia and the persistent activation of cancer-associated fibroblasts (CAFs) (91). These mechanisms collectively contribute to tumour progression, therapeutic resistance, local recurrence, and poorer clinical outcomes in areca nut-associated OSCC (91).

Although epithelial EMT and fibroblast activation share several upstream signalling mediators, including TGF-β, ZEB1, Snail, Twist, and inflammatory cytokines, fibroblast-to-myofibroblast differentiation represents a distinct biological process and should not be interpreted as epithelial–mesenchymal transition (92).

## 6 Inflammation and EMT crosstalk in OSCC

Chronic inflammation and EMT mutually reinforce each other, facilitating tumour invasion, metastasis, and resistance to therapy (92). In OSCC, inflammatory cytokines in the TME and immune cells, such as macrophages and lymphocytes, are the principal inducers of EMT and sustain tumour progression (93). The cytokines TGF-β, TNF-α, IL-1, IL-6, IL-8, CCL5, and IL-17A promote EMT by activating EMT-related transcription factors, including Snail, Slug, TWIST, and ZEB, through signalling pathways such as Smad, NF-κB, and Signal Transducer and Activator of Transcription 3 (STAT3) (94, 95). This activation leads to a reduction in E-cadherin levels and an increase in vimentin and N-cadherin levels, thereby promoting EMT and stem-like characteristics (96). In OSCC, microenvironmental CCL5 and IL-6, including IL-6 derived from M1-like tumour-associated macrophages (TAMs), further enhance EMT and lymph node metastasis (97, 98).

Inflammatory cytokines, including TGF-β, TNF-α, IL-1, and IL-6, activate the NF-κB and Smad signalling pathways, collectively inducing EMT-transcription factors (EMT-TFs) and EMT itself (99). In a Ras/TGF-β breast cancer model, NF-κB is essential for both the induction and maintenance of EMT; inhibiting NF-κB effectively prevents EMT and eliminates metastasis (100). TGF-β promotes EMT through the NF-κB/NOX4/ROS axis, where NF-κB is positioned upstream of the ROS-producing NOX4, thereby enhancing cellular migration and proliferation (101). The combination of platelet-derived TGF-β and direct cellular contact synergistically activates the TGF-β/Smad and NF-κB pathways in tumour cells, leading to EMT-like alterations and increased metastatic potential (102). Inhibition of either NF-κB or platelet-derived TGF-β results in a reduction in lung metastases (103).

Negative feedback mechanisms mitigate the effects of TGF-β, which induces the long noncoding RNA NKILA, inhibiting NF-κB signalling and attenuating TGF-β-mediated EMT and metastasis (104). Nevertheless, inflammatory cytokines and EMT establish self-reinforcing feedback loops, whereby cytokines activate NF-κB, STAT3, and Smad pathways. This activation triggers EMT-TFs and epigenetic regulators, thereby stabilising EMT and CSC phenotypes, while amplifying cytokine production (105). In OSCC specific circuits, including IL-6/STAT3/miR-34a, IL-6/STAT3/THBS1, and IL-6–STAT3–C/EBPβ–IL-6, enhance EMT, invasion, metastasis, and sustained TAM activation, thereby perpetuating a pro-tumourigenic inflammatory microenvironment (91, 106, 107).

Inflammation induced EMT directly facilitates invasion, lymphatic and distant metastasis, angiogenesis, immune evasion, and chemoresistance in various cancers, including OSCC (108). In a syngeneic mouse model, OSCC cells with high metastatic potential exhibited enhanced EMT, and inflammatory cytokines CCL5 and IL-6 further augmented EMT and cervical lymph node metastasis (109). TAM derived IL-6 enhances invasion, migration, xenograft formation, and mesenchymal/stem-like phenotypes in OSCC through EMT (110). Systemically, platelet-driven TGF-β/NF-κB activation during detachment of tumour cells from primary tumour and circulation further exacerbate locoregional and distant metastasis (111).

## 7 Mechanisms of treatment resistance

### 7.1 Chemotherapy resistance mechanisms

The evidence connecting areca nut exposure to therapeutic resistance varies and should be interpreted within the context of specific experiments (109). Direct evidence is mainly obtained from models of oral epithelial and OSCC cells that have been chronically exposed to arecoline or areca nut extract, which show decreased sensitivity to chemotherapeutic agents (109). Conversely, several mechanisms, such as hypoxia-mediated radiotherapy resistance, altered DNA damage responses, and TME-mediated therapeutic adaptation, have been primarily identified in OSCC or other malignancies without specific stratification based on areca nut exposure (110). These mechanisms are thus discussed as biologically plausible pathways that may interact with areca nut-induced inflammatory and EMT programmes, rather than as definitively established characteristics of areca nut-associated OSCC (112).

Chemoresistance presents a significant clinical challenge in OSCC (112). Evidence from cell-based studies indicates that prolonged exposure to areca nut alkaloids, especially arecoline, may lead to decreased sensitivity to chemotherapeutic agents, such as cisplatin and fluorouracil (112). The emergence of chemotherapeutic resistance entails cellular adaptations at physiological, genomic, and epitranscriptomic levels (112).

Long-term exposure to arecoline results in cisplatin and fluorouracil resistance by causing overexpression of ATP Binding Cassette Subfamily G (ABCG2) proteins, which affects drug efflux pumps. OSCC also exhibits enhanced drug efflux and sequestration, along with upregulation of ATP-binding cassette transporters that are actively involved in the removal of chemotherapeutic agents, ultimately preventing them from reaching their target sites and thus reducing intracellular concentrations (112). The interaction between arecoline and intracellular signalling cascades leads to the suppression of pro-apoptotic proteins, such as Bcl-2 associated X protein (BAX), and upregulation of anti-apoptotic proteins, such as members of the B-cell lymphoma -2 (Bcl-2) family (112). This change promotes cell cycle continuation through Methyltransferase like 3 (METTL3) stabilized CDK1 (8, 11).

Recent evidence suggests that RNA N6-methyladenosine (m6A) plays a key role at the epitranscriptomic level (113). Arecoline exposure causes upregulation of methyltransferase METTL3, which stabilises survival-related mRNAs such as CDK1, in an m6A-dependent manner, ensuring cell cycle progression, bypassing drug induced cell cycle arrest, and ultimately producing cisplatin resistance (113).

Long-term areca nut chewing induces metabolic modification of cells and contributes to the liberation of ROS, which triggers protective autophagy (111). This process functions as a protective mechanism for cancer cells by allowing the repair and recycling of damaged organelles and proteins, thereby mitigating the cytotoxic effects of cisplatin treatment. Recent studies have shown that the reversal of this resistant phenotype can be achieved by inhibiting autophagic flux (114).

Large scale genomic surveys have indicated that habitual chewing of areca nut results in frequent changes in p53 and Hippo signalling networks, including mutations in TP53 and Conserved Helix Loop Helix Ubiquitous Kinase (CHUK), along with copy number gains of MAP3K13 and Fas Associated Protein with Death Domain (FADD), thus creating a cellular microenvironment that promotes cancer cell proliferation and resistance to chemotherapeutic agents (113, 114).

### 7.2 Radiotherapy resistance mechanisms

The interaction between the TME and genomic stability is the reason for the development of radiotherapy resistance in OSCC (115). Long-term chewing of areca nuts not only enhances exposure to carcinogenic agents, such as arecoline or arecaidine, but also exerts selective pressure on the oral mucosa. This causes changes in the microenvironment and DNA damage (115). This change in the microenvironment is favourable for the survival of cell populations that have undergone cellular mutations involving those with DNA repair machinery changes, especially involving the resolution of double-strand breaks induced by ionising radiation (116).

Tumour hypoxia is a recognised factor contributing to radiotherapy resistance in OSCC. Chronic oxidative stress and microenvironmental changes induced by areca nut exposure may promote hypoxia-related signalling pathways. However, direct evidence linking areca nut exposure to increased radiotherapy resistance remains insufficient (116). Oxygen behaves as a radiosensitizer and reacts with radiation-induced free radicals, making DNA damage permanent and, thus making the cells susceptible to destruction by radiation (117). Hence, in hypoxic states, tumour cells are resistant to radiation-induced cellular damage. The hypoxic microenvironment is stabilised by the ROS/HIF-1α axis, which prevents the degradation of HIF-1α, which in turn activates transcriptional factors promoting anaerobic environment and cell survival (116, 117).

Multiomics profiling has revealed that areca nut-associated tumours show increased sprouting angiogenesis, which is structurally dysfunctional and leaky (116). This leads to the formation of a microenvironment that contains cells that are treatment resistant in a hypoxic environment, thus being fully protected from radiotherapy. This altered microenvironment prevents the uniform delivery of oxygen and therapeutic agents, and the acidosis produced further inhibits the effectiveness of radiation induced ionisations (116, 117).

Chronic inflammatory fields produced by areca nut extracts promote the infiltration of specialised stromal cells that produce protective growth factors, stimulating the rapid repopulation of tumour cells, thus ensuring a malignant field that can withstand and recover from therapeutic doses of radiation therapy (118).

### 7.3 EMT and resistance

Chronic exposure to arecoline triggers the development of epithelial-to-mesenchymal transition (105). This change occurs due to the downregulation of E-cadherin and upregulation of N-cadherin, promoting mesenchymal trans differentiation which increases cell motility, loss of cell adhesion, and plasticity, thus increasing the risk of cancer spread (107). EMT acts as a bridge between tumour progression and treatment resistance (114).

This transition is also associated with cancer stemness. Arecoline exposure has been associated with the enrichment of ALDH-1 positive and CD44-positive stem cell populations (107). These stem cells can remain in a quiescent state, and because the treatment regimen for OSCC focuses on rapidly dividing cells, these stem cells are often protected from standard treatment agents (110). The molecular maintenance of the state is managed by a group of transcription factors, including Oct4, NANOG, and SOX2 (113, 114).

Areca nut exposure causes the upregulation of these factors due to the dysregulation of miRNAs, such as the loss of miR-145 in areca-exposed cells, directly allowing the stabilisation of Oct4 and Sox2 mRNAs (110). This axis is considered an important contributor to chemoresistance in OSCC (110). The EMT process induces the expression of anti-apoptotic proteins and drug transporters, creating a double-lock mechanism of resistance (112). The CSCs produced can cause disease relapse and secondary tumours (118).

### 7.4 Contribution of inflammatory pathways

The inflammatory pathway observed in OSCC due to areca nut chewing is chronic and not merely a byproduct of tumourigenesis (114). Chronic inflammatory signalling is a significant factor contributing to therapeutic resilience, as it coordinates various signalling pathways that enhance cancer cell survival and adaptation (119). Arecoline exposure, along with other carcinogenic extracts of areca nut, induces mechanical and chemical stress in the oral mucosa (114). This stress damages DNA and promotes cancer stemness and treatment resistance (117). Chronic inflammation produces genomic instability and tumour initiation through epigenetic changes and mutagen production (114).

Recent multimodal profiling performed by Shih-Chi Su et al. in patients with OSCC showed a specific boost in signals, such as CHUK and MAP3K13. These molecules activate the NF-κB pathway, promoting cell survival and leading to the production of anti-apoptotic proteins, such as the Bcl-2 family, even after exposure to chemotherapeutic agents or radiation (120). ROS generated during areca nut chewing induce adaptive antioxidant responses and activate pro-survival signalling pathways, thus enhancing reduced therapeutic responsiveness (117).

The inflammatory niche formed produces chemoattractive molecules and facilitates the recruitment of supportive stromal cells (92). Chronic inflammation triggers the release of chemokines that attract CAFs and immunosuppressive cells to the TME (14). These molecules alter the TME and ensure that the malignant epithelium is surrounded by a dense extracellular matrix that acts as a physical barrier to therapeutic agents (114, 116).

The inflammatory microenvironment facilitates immune evasion by forming an immune-suppressed niche phenotype and reprogramming local immune cell populations through a dendritic cell regulatory programme (121). The inflamed niche converts cells into a tolerogenic state, causing immunosuppression. This produces an immunodeficient state characterised by the exclusion of T-effector cells from the tumour core (121). This immune evasion is reinforced by CAFs, which secrete growth factors to stimulate CSC phenotypes, thereby promoting tumour resilience and recurrence (122).

## 8 Therapeutic targeting opportunities

### 8.1 Targeting inflammatory pathways

Chronic exposure to areca/betel leads to the generation of ROS, inflammation, and EMT, forming a network of pathways driven by NF-κB, COX-2, and cytokines that facilitate OSF and OSCC progression (95). Although most evidence remains preclinical or conceptual, these pathways represent rational targets for intervention, albeit not yet established as standard therapies for betel chewing (100). Arecoline-induced ROS, TGF-β1, and inflammatory cytokines contribute to EMT and malignant transformation. Furthermore, elevated membranous Notch1 expression has been linked to the progression from oral leucoplakia to OSCC, underscoring its involvement in oral carcinogenesis (123). In experimental models, antioxidants and targeted inhibitors of EMT inducers have been shown to mitigate oral cancer progression (124). Reviews of betel-associated cancer highlight the potential of anti-inflammatory drugs as chemopreventive agents in patients who are disease-free after surgery. Areca nut extract significantly activates NF-κB in oral keratinocytes, and the inhibition of NF-κB effectively suppresses ANE-induced COX-2 upregulation (125).

Areca activates NF-κB-linked pathways, including EGFR, MAPK, and PI3K/Akt, which promote MMP-9 expression, invasion, and EMT (108). Although general NF-κB-targeted strategies, such as IKK inhibitors, proteasome inhibitors, nuclear translocation/DNA-binding inhibitors, tyrosine kinase inhibitors (TKIs), and non-coding RNAs (ncRNAs), are being actively developed for oncology and inflammatory diseases, they have not been specifically evaluated in patients with areca nut chewing habit associated OSCC (126). miRNA-based modulation, exemplified by miR-107’s inhibition of ERK/NF-κB and EMT in OSCC, indicates potential additional NF-κB-directed strategies for OSCC treatment (108). Areca nut extract and arecoline stimulate COX-2 and PGE_2_ production in oral keratinocytes and carcinoma cells, contributing to inflammation and immune evasion (127). A review highlighted that celecoxib and curcumin have been proposed as potential chemopreventive strategies based primarily on preclinical and mechanistic evidence for betel quid-associated cancer (108). Another review suggested that inhibiting IL-1α or COX reduces tumour development in experimental models, while elevated PGE_2_ levels facilitate immune evasion, thereby supporting COX-2 as a viable chemopreventive or adjunctive target (128).

### 8.2 Targeting EMT

The strategy for targeting EMT in malignancies associated with Areca nut primarily involves inhibiting TGF-β signalling and employing agents that counteract EMT-like alterations induced by inflammation and Areca nut exposure (74). Arecoline activates the TGF-β/Activin Receptor-Like Kinase 5 (ALK5)–Smad2/3 and TGF-β2/THBS1 pathways in oral epithelial cells, establishing TGF-β as a pivotal factor in the development of fibrosis and EMT in OSF and related lesions (129). Direct inhibitors of TGF-β receptor I, such as SB525334, GW788388, and A83-01, effectively block TGF-β1-induced EMT by reversing morphological transitions, migration, invasion, mesenchymal marker upregulation, collagen synthesis, and Smad2/3 phosphorylation in epithelial cells (130). These inhibitors have also demonstrated efficacy in reducing fibrosis *in vivo*, while maintaining barrier integrity (130). Additionally, ligand-trap biologics that sequester all TGF-β isoforms (TβRI-TβRII-Fc) inhibit TGF-β-induced EMT in oral cancer cells and suppress tumour growth and angiogenesis in xenograft models (131). This is achieved through the downregulation of HB-EGF, IL-1β, and Epiregulin (EREG) in the TME, suggesting a promising approach for controlling EMT at the TME level in OSCC (132).

As comprehensive strategies for reversing EMT, both Natural and small-molecule agents targeting EMT signalling pathways have been identified as comprehensive strategies for reversing EMT (133). These include polyphenols such as EGCG, resveratrol, and curcumin, which downregulate TGF-β1, collagen, and inflammatory mediators in areca-induced OSF models (134). Additionally, plant compounds that inhibit pathways such as TGF-β/Smads, Wnt/β-catenin, NF-κB, PI3K/Akt, and EMT transcription factors (Snail, Twist, and Zeb) are of interest (135). Multi-target kinase inhibitors, such as nintedanib, which suppress TGF-β-driven EMT via Smad2/3, provide mechanistic support for targeting TGF-β-driven EMT. However, their therapeutic efficacy in areca nut-associated OSCC has not yet been established (136). However, the clinical translation of these strategies in areca nut chewers remains limited and predominantly preclinical (136).

### 8.3 Combined therapeutic approaches

Employing combined therapeutic strategies that concurrently address inflammation and EMT is a logical approach to disrupt the interconnected pathogenic network instigated by chronic exposure to areca nut. Areca-induced ROS and inflammatory signalling pathways, including NF-κB, COX-2, and cytokines, perpetuate a tumour-promoting microenvironment and enhance TGF-β-mediated EMT and fibrosis in HNSCC (44, 137–140). EMT programmes exacerbate invasive behaviour and immune evasion, as exemplified by PGE_2_ (138). Therefore, the simultaneous targeting of inflammatory pathways using selective COX-2 inhibitors, NF-κB pathway modulators, or anti-cytokine biologics alongside EMT-focused agents, such as TGF-βRI inhibitors, ligand-trap biologics, multi-kinase inhibitors, or small molecules/miRNA modulators that reverse EMT transcriptional programmes, is anticipated to be synergistic (141–143). Preclinical studies suggest that combined targeting of inflammatory and EMT pathways may reduce collagen deposition, EMT marker expression, invasion, and tumour growth (144). However, validation in clinically relevant areca nut-associated OSCC models is necessary (26).

## 9 Future research directions

Effective translation necessitates the selection of patients based on biomarkers and careful optimisation of dosage and sequencing to reduce overlapping toxicities. Potential biomarkers include COX-2/PGE_2_, pSmad2/3, nuclear NF-κB, EMT gene signatures, and immune profiling from tissue or liquid samples. Adaptive biomarker-stratified trial designs can assess matched anti-inflammatory and anti-EMT regimens across inflammation-dominant, EMT-dominant, and mixed phenotypes. Additionally, low-cost saliva/plasma assays and regional capacity building are crucial for equitable implementation in high-burden chewing tobacco populations. Ultimately, the integration of anti-inflammatory and anti-EMT modalities guided by molecular phenotyping and iterative biomarker endpoints offers a viable approach to enhance chemoprevention and adjuvant therapy for areca-associated oral disease.

Future research should prioritise the development of areca-relevant preclinical models, such as chronic arecoline/areca nut extract exposed organoids, OSF rodent models, and patient derived xenografts. These models are essential for evaluating safety and efficacy prior to human trials. Translational studies should implement adaptive biomarker stratified trial designs that align anti-inflammatory interventions targeting COX-2/NF-κB/cytokine pathways with anti-EMT strategies, including TGF-βRI inhibitors, ligand traps, and epigenetic or miRNA based modulators, to address inflammation or EMT dominant phenotypes. Intermediate endpoints, such as reduced PGE_2_ levels, attenuation of EMT signatures, and altered immune infiltration, may facilitate earlier decision making. Simultaneously, public health initiatives, including regulations, cessation support, and culturally tailored education, are crucial for reducing areca nut exposure at the population level. Building capacity for affordable molecular diagnostics and ensuring inclusive recruitment in high burden regions are vital for equitable implementation. Longitudinal prospective studies are necessary to delineate biomarker trajectories in areca nut chewing behaviour associated OSF field cancerisation, validate predictors of malignant transformation, and assess whether biomarker guided interventions enhance clinical outcomes.

## 10 Conclusion

Areca nut chewing facilitates oral carcinogenesis through a complex pathogenic axis encompassing chronic inflammation, oxidative stress, fibrosis, EMT, cancer stemness, immune evasion, and resistance to chemotherapy and radiotherapy. These processes are mechanistically interconnected via inflammatory mediators such as NF-κB, COX-2, and cytokine networks, as well as EMT-associated pathways, including TGF-β, Wnt/β-catenin, and Notch. Collectively, these factors transform a chronically injured oral mucosal field into a tumour-promoting microenvironment, thereby facilitating the progression of OMPDs to aggressive OSCC. Clinically, this underscores the importance of areca nut cessation, early identification of high-risk lesions, and biomarker-guided targeting of inflammatory and EMT-related pathways as pivotal strategies for prevention and adjunctive therapy of OSCC. Future research should aim to validate these pathways in longitudinal human cohorts. Additionally, it is necessary to develop practical biomarkers capable of stratifying the risk, monitoring disease progression, and guiding treatment responses in populations exposed to areca nuts.

## References

[1] Su SC Lin CW Chen MK Lee YC Su CW Bai S . Multimodal profiling of oral squamous cell carcinoma identifies genomic alterations and expression programmes associated with betel quid chewing. Neoplasia (United States). (2025) 68:101218. doi: 10.1016/j.neo.2025.101218 40784158 PMC12357113

[2] Warnakulasuriya S Chen THH . Areca nut and oral cancer: Evidence from studies conducted in humans. J Dent Res. (2022) 101:1139–46. doi: 10.1177/00220345221092751 35459408 PMC9397398

[3] Li YC Cheng AJ Lee LY Huang YC Chang JTC . Multifaceted mechanisms of areca nuts in oral carcinogenesis: The molecular pathology from precancerous condition to Malignant transformation. J Cancer. (2019) 10:4054–62. doi: 10.7150/jca.29765 31417650 PMC6692602

[4] Kamal MV Damerla RR Parida P Chakrabarty S Rao M Kumar NA . Antiapoptotic PON2 expression and its clinical implications in locally advanced oral squamous cell carcinoma. Cancer Sci. (2024) 115:2012–22. doi: 10.1111/cas.16170 38602182 PMC11145147

[5] Xu W Li C Liu Q Liu W Wang X . A scientometric study of oral cancer research in South and Southeast Asia with emphasis on risk factors control. J Dent Sci. (2024) 19:2157–62. doi: 10.1016/j.jds.2024.03.014 39347074 PMC11437299

[6] Albert M-S Ko PWWWTLCHL . Betel-quid addictive use disorders and Oral potentially Malignant disorders and Oral cancer in south, southeast, and East Asia: A systematic review and meta-analysis. 31(5):1517–30. doi: 10.1080/14786419.2026.2615746 39164987

[7] Senevirathna K Pradeep R Jayasinghe YA Jayawickrama SM Illeperuma R Warnakulasuriya S . Carcinogenic effects of areca nut and its metabolites: A review of the experimental evidence. Clinics Pract. (2023) 13:326–46. doi: 10.3390/clinpract13020030 36961055 PMC10037666

[8] Wang C Kadigamuwa C Wu S Gao Y Chen W Gu Y . RNA N6-methyladenosine (m6A) methyltransferase-like 3 facilitates tumourigenesis and cisplatin resistance of arecoline-exposed oral carcinoma. Cells. (2022) 11:3605. doi: 10.3390/cells11223605 36429032 PMC9688745

[9] Gunjal S Pateel DGS Yang YH Doss JG Bilal S Maling TH . An overview on betel quid and areca nut practice and control in selected Asian and South East Asian countries. Subst Use Misuse. (2020) 55:1533–49. doi: 10.1080/10826084.2019.1657149 32569533

[10] Kumar A Oswal K Singh R Kharodia N Pradhan A Sethuraman L . Assessment of areca nut use, practice and dependency among people in Guwahati, Assam: A cross-sectional study. Ecancermedicalscience. (2021) 15:1198. doi: 10.3332/ECANCER.2021.1198 33889207 PMC8043683

[11] Sharma M Sarode SC Sarode G Radhakrishnan R . Areca nut-induced oral fibrosis – Reassessing the biology of oral submucous fibrosis. J Oral Biosci. (2024) 66:320–8. doi: 10.1016/j.job.2024.02.005 38395254

[12] Gocol H Zeng JH Chang S Koh BY Nguyen H Cirillo N . A critical interpretive synthesis of the role of arecoline in oral carcinogenesis: Is the local cholinergic axis a missing link in disease pathophysiology? Pharmaceuticals. (2023) 16:1684. doi: 10.3390/ph16121684 38139811 PMC10748297

[13] Ko AMS Tu HP Ko YC . Systematic review of roles of arecoline and arecoline N-oxide in oral cancer and strategies to block carcinogenesis. Cells. (2023) 12:1208. doi: 10.3390/cells12081208 37190117 PMC10137008

[14] Gok Yavuz B Gunaydin G Gedik ME Kosemehmetoglu K Karakoc D Ozgur F . Cancer associated fibroblasts sculpt tumour microenvironment by recruiting monocytes and inducing immunosuppressive PD-1+ TAMs. Sci Rep. (2019) 9:3172. doi: 10.1038/s41598-019-39553-z 30816272 PMC6395633

[15] Peralta-Mamani M Terrero-Pérez Á Tucunduva RMA Rubira CMF Santos PS Honório HM . Occurrence of field cancerization in clinically normal oral mucosa: A systematic review and meta-analysis. Arch Oral Biol. (2022) 145:105544. doi: 10.1016/j.archoralbio.2022.105544 36126567

[16] Puspasari K Pasaribu TAS Surboyo MDC Ayuningtyas NF Santosh ABR Ernawati DS . Oral field cancerization: Genetic profiling for a prevention strategy for oral potentially Malignant disorders. Dental J. (2023) 56:189–96. doi: 10.20473/j.djmkg.v56.i3.p189-196

[17] Kazemi K Fadl A Sperandio FF Leask A . The areca nut and oral submucosal fibrosis: A narrative review. Dentistry J. (2025) 13:364. doi: 10.3390/dj13080364 40863067 PMC12385254

[18] Sharan RN Mehrotra R Choudhury Y Asotra K . Association of betel nut with carcinogenesis: Revisit with a clinical perspective. PloS One. (2012) 7:e42759. doi: 10.1371/journal.pone.0042759 22912735 PMC3418282

[19] Faouzi M Neupane RP Yang J Williams P Penner R . Areca nut extracts mobilize calcium and release pro-inflammatory cytokines from various immune cells. Sci Rep. (2018) 8:4215. doi: 10.1038/s41598-017-18996-2 29348572 PMC5773534

[20] Wang CC Lin HL Wey SP Jan TR . Areca-nut extract modulates antigen-specific immunity and augments inflammation in ovalbumin-sensitized mice. Immunopharmacol Immunotoxicol. (2011) 33:315–22. doi: 10.3109/08923973.2010.507208 20698815

[21] Cai C Guan L Wang C Hu R Ou L Jiang Q . The role of fibroblast-neutrophil crosstalk in the pathogenesis of inflammatory diseases: a multi-tissue perspective. Front Immunol. (2025) 16. doi: 10.3389/fimmu.2025.1588667 40486514 PMC12140995

[22] Laing AG Riffo-Vasquez Y Sharif-Paghaleh E Lombardi G Sharpe PT . Immune modulation by apoptotic dental pulp stem cells *in vivo*. Immunotherapy. (2018) 10:201–11. doi: 10.2217/imt-2017-0117 29370720 PMC5810843

[23] Rani P Shivaprasad Mishra M . Emerging insights into supari (areca nut) toxicity: a review of current evidence on oral and general health. Toxin Rev. (2025) 44:383–96. doi: 10.1080/15569543.2025.2538139 37339054

[24] Huang Z He Z Dong L Li D Chen F Hu X . Microbial signatures of areca nut chewing: Bridging the gap between 2 oral dysbiosis and submucous fibrosis. Available online at: https://ssrn.com/abstract=5294411 (Accessed June 6, 2026). 10.1016/j.phymed.2025.157227 40934758

[25] Huang G Zeng D Liang T Liu Y Cui F Zhao H . Recent advance on biological activity and toxicity of arecoline in edible areca (betel) nut: A review. Foods. (2024) 13:3825. doi: 10.3390/foods13233825 39682896 PMC11639887

[26] Yu N Cai W Zhang C Cai Q Zhang Z Hu Y . The multistep progression of areca nut-induced oral cancer: a mechanistic roadmap from pathogenesis to precision therapy. Life Med. (2026) 5:lnag011. doi: 10.1093/lifemedi/lnag011 42100377 PMC13148401

[27] Poligone B Baldwin AS . Positive and negative regulation of NF-κB by COX-2. Roles of different prostaglandins. J Biol Chem. (2001) 276:38658–64. doi: 10.1074/jbc.M106599200 11509575

[28] Lin S Lu S Lee S Lin C Chen C Chang K . Areca (betel) nut extract activates mitogen‐activated protein kinasesand NF‐κB in oral keratinocytes. Int J Cancer. (2005) 116:526–35. doi: 10.1002/ijc.21104 15825184

[29] Wang TH Shen YW Chen HY Chen CC Lin NC Shih YH . Arecoline induces ROS accumulation, transcription of proinflammatory factors, and expression of KRT6 in oral epithelial cells. Biomedicines. (2024) 12:412. doi: 10.3390/biomedicines12020412 38398015 PMC10887121

[30] Li P Zhang S Mo Y Zhang L Wang Y Xiong F . Long non-coding RNA expression profiles and related regulatory networks in areca nut chewing-induced tongue squamous cell carcinoma. Oncol Lett. (2020) 20:4863–74. doi: 10.3892/OL.2020.12165 33093911 PMC7573881

[31] Murakami E Shionoya T Komenoi S Suzuki Y Sakane F . Cloning and characterization of novel testis-specific diacylglycerol kinase η splice variants 3 and 4. PloS One. (2016) 11:e0151885. doi: 10.1371/journal.pone.0162997 27643686 PMC5028035

[32] Chiang SL Chen PH Lee CH Ko AMS Lee KW Lin YC . Up-regulation of inflammatory signalling by areca nut extract and role of cyclooxygenase-2 -1195G>A polymorphism reveal risk of oral cancer. Cancer Res. (2008) 68:8489–98. doi: 10.1158/0008-5472.CAN-08-0823 18922923

[33] Kim D Illeperuma RP Kim J . The protective effect of antioxidants in areca nut extract-induced oral carcinogenesis. Asian Pacific J Cancer Prev. (2020) 21:2447–52. doi: 10.31557/APJCP.2020.21.8.2447 32856877 PMC7771929

[34] Muthupalani S Annamalai D Feng Y Ganesan SM Ge Z Whary MT . IL-1β transgenic mouse model of inflammation driven oesophageal and oral squamous cell carcinoma. Sci Rep. (2023) 13:14140. doi: 10.1038/s41598-023-39907-8 37543673 PMC10404242

[35] Ren Q Qu L Yuan Y Wang F . Natural modulators of key signalling pathways in skin inflammageing. Clinical Cosmetic Investigational Dermatol. (2024) 17:2967–88. doi: 10.2147/CCID.S502252 39712942 PMC11663375

[36] Chang MC Chan CP Chen YJ Hsien HC Chang YC Yeung SY . Areca nut components stimulate ADAM17, IL-1`, PGE2 and 8-isoprostane production in oral keratinocyte: role of reactive oxygen species, EGF and JAK signalling. In: Oncotarget Albany: Impact Journals (2016). Available online at: https://www.impactjournals.com/oncotarget (Accessed June 6, 2026). 10.18632/oncotarget.7621 PMC4941357 26919242

[37] Zhang P Chua NQE Dang S Davis A Chong KW Prime SS . Molecular mechanisms of Malignant transformation of oral submucous fibrosis by different betel quid constituents—does fibroblast senescence play a role? Int J Mol Sci. (2022) 23:1637. doi: 10.3390/ijms23031637 35163557 PMC8836171

[38] Ji WT Yang SR Chen JYF Cheng YP Lee YR Chiang MK . Arecoline downregulates levels of p21 and p27 through the reactive oxygen species/mTOR complex 1 pathway and may contribute to oral squamous cell carcinoma. Cancer Sci. (2012) 103:1221–9. doi: 10.1111/j.1349-7006.2012.02294.x 22469187 PMC7659358

[39] Ezhilarasan D Jayanth Kumar V . Toxicological impacts of arecoline and tobacco in oral submucous fibrosis: Mechanisms and therapeutic insights. J Appl Toxicol. (2025) 45:2498–511. doi: 10.1002/jat.4877 40763929

[40] Bonnici L Suleiman S Schembri-Wismayer P Cassar A . Targeting signalling pathways in chronic wound healing. Int J Mol Sci. (2024) 25:50. doi: 10.3390/ijms25010050 38203220 PMC10779022

[41] Illeperuma RP Kim DK Park YJ Son HK Kim JY Kim J . Areca nut exposure increases secretion of tumour-promoting cytokines in gingival fibroblasts that trigger DNA damage in oral keratinocytes. Int J Cancer. (2015) 137:2545–57. doi: 10.1002/ijc.29636 26076896 PMC4744697

[42] Tang J Liu J Zhou Z Cui X Tu H Jia J . Oral submucous fibrosis: pathogenesis and therapeutic approaches. Int J Oral Sci. (2025) 17:8. doi: 10.1038/s41368-024-00344-6 39890798 PMC11785813

[43] Kumari P Debta P Dixit A . Oral potentially Malignant disorders: Aetiology, pathogenesis, and transformation into oral cancer. Front Pharmacol. (2022) 13. doi: 10.3389/fphar.2022.825266 35517828 PMC9065478

[44] Kamal MV Palod A Parida P Pai A Sharan K Belle VS . Modulation of prostaglandin-endoperoxide synthase-2 (PTGS2) mediated VEGF-signalling pathway by oral metronomic chemotherapy in locally advanced oral squamous cell carcinoma: A brief report. Cancer Chemother Pharmacol. (2025) 95:96. doi: 10.1007/s00280-025-04819-z 41045400

[45] Kamal MV Damerla RR Parida P Rao M Belle VS Dikhit PS . Expression of PTGS2 along with genes regulating VEGF signalling pathway and association with high-risk factors in locally advanced oral squamous cell carcinoma. Cancer Med. (2024) 13:e6986. doi: 10.1002/cam4.6986 38426619 PMC10905678

[46] Kamal MV Prabhu K Sharan K Pai A Chakrabarty S Damerla RR . Investigation of the molecular mechanisms of paraoxonase-2 mediated radiotherapy and chemotherapy resistance in oral squamous cell carcinoma. Clin Transl Sci. (2025) 18:e70201. doi: 10.1111/cts.70201 40134131 PMC11936840

[47] Filip-Psurska B Zachary H Strzykalska A Wietrzyk J . Vitamin D, Th17 lymphocytes, and breast cancer. Cancers. (2022) 14:3649. doi: 10.3390/cancers14153649 35954312 PMC9367508

[48] Cui X Liu S Song H Xu J Sun Y . Single-cell and spatial transcriptomic analyses revealing tumour microenvironment remodelling after neoadjuvant chemoimmunotherapy in non-small cell lung cancer. Mol Cancer. (2025) 24:87. doi: 10.1186/s12943-025-02287-w 40205583 PMC11980172

[49] Park MN Kim M Lee S Kang S Ahn CH Tallei TE . Targeting redox signalling through exosomal microRNA: Insights into tumour microenvironment and precision oncology. Antioxidants. (2025) 14:501. doi: 10.3390/antiox14050501 40427384 PMC12108341

[50] Athukorala IA Tilakaratne WM Jayasinghe RD . Areca nut chewing: Initiation, addiction, and harmful effects emphasizing the barriers and importance of cessation. J Addict. (2021) 2021:1–9. doi: 10.1155/2021/9967097 34123457 PMC8192186

[51] Wang L Liu X Chen M Hu Y Zhu L . Oral squamous cell carcinoma‐risk factors, diagnosis, treatment, and prevention. Cancer Nexus. (2026) 2:70022. doi: 10.1002/cnx2.70022 41531421

[52] Ling Z Cheng B Tao X . Epithelial‐to‐mesenchymal transition in oral squamous cell carcinoma: Challenges and opportunities. Int J Cancer. (2021) 148:1548–61. doi: 10.1002/ijc.33352 33091960

[53] Rai H Ahmed J . N-cadherin: A marker of epithelial to mesenchymal transition in tumour progression. Internet J Oncol. (2014) 10:1–7.

[54] Puneeta N Santosh T Mishra I Gaikwad P Sahu A . Evaluation of e-cadherin and vimentin expression for different grades of oral epithelial dysplasia and oral squamous cell carcinoma - An immunohistochemical study. J Oral Maxillofac Pathol. (2022) 26:285. doi: 10.4103/jomfp.JOMFP_166_20 35968190 PMC9364642

[55] Guarino M Rubino B Ballabio G . The role of epithelial-mesenchymal transition in cancer pathology. Pathology. (2007) 39:305–18. doi: 10.1080/00313020701329914 17558857

[56] Kim SA Inamura K Yamauchi M Nishihara R Mima K Sukawa Y . Loss of CDH1 (E-cadherin) expression is associated with infiltrative tumour growth and lymph node metastasis. Br J Cancer. (2016) 114:199–206. doi: 10.1038/bjc.2015.347 26742007 PMC4815802

[57] Costa LCMC Leite CF Cardoso SV Loyola AM de Faria PR Souza PEA . Expression of epithelial-mesenchymal transition markers at the invasive front of oral squamous cell carcinoma. J Appl Oral Sci. (2015) 23:169–78. doi: 10.1590/1678-775720140187 26018309 PMC4428462

[58] Lorenzo-Pouso AI Silva F Pérez-Jardón A Chamorro-Petronacci CM Oliveira-Alves MG Álvarez-Calderón-Iglesias Ó . Overexpression of E-cadherin is a favourable prognostic biomarker in oral squamous cell carcinoma: A systematic review and meta-analysis. Biology. (2023) 12:239. doi: 10.3390/biology12020239 36829516 PMC9953277

[59] Balasundaram P Singh MK Dinda AK Thakar A Yadav R . Study of β-catenin, E-cadherin and vimentin in oral squamous cell carcinoma with and without lymph node metastases. Diagn Pathol. (2014) 9:145. doi: 10.1186/1746-1596-9-145 25047112 PMC4223686

[60] Zhou B Xiang J Jin M Zheng X Li G Yan S . High vimentin expression with E-cadherin expression loss predicts a poor prognosis after resection of grade 1 and 2 pancreatic neuroendocrine tumours. BMC Cancer. (2021) 21:1230. doi: 10.1186/s12885-021-08062-6 33789624 PMC8010952

[61] Gravdal K Halvorsen OJ Haukaas SA Akslen LA . A switch from E-cadherin to N-cadherin expression indicates epithelial to mesenchymal transition and is of strong and independent importance for the progress of prostate cancer. Clin Cancer Res. (2007) 13:7003–11. doi: 10.1158/1078-0432.CCR-07-1263 18056176

[62] Ghantous Y Mozalbat S Nashef A Abdol-Elraziq M Sudri S Araidy S . EMT dynamics in lymph node metastasis of oral squamous cell carcinoma. Cancers (Basel). (2024) 16:1185. doi: 10.3390/cancers16061185 38539520 PMC10968900

[63] De Wever O Pauwels P De Craene B Sabbah M Emami S Redeuilh G . Molecular and pathological signatures of epithelial-mesenchymal transitions at the cancer invasion front. Histochem Cell Biol. (2008) 130:481–94. doi: 10.1007/s00418-008-0464-1 18648847 PMC2522326

[64] Tan Y Wang Z Xu M Li B Huang Z Qin S . Oral squamous cell carcinomas: State of the field and emerging directions. Int J Oral Sci. (2023) 15:43. doi: 10.1038/s41368-023-00249-w 37736748 PMC10517027

[65] Fuxe J Vincent T Garcia de Herreros A . Transcriptional crosstalk between TGFβ and stem cell pathways in tumour cell invasion: Role of EMT promoting Smad complexes. Cell Cycle. (2010) 9:2363–74. doi: 10.4161/cc.9.12.12050 20519943

[66] Meng L Zhao Y Bu W Li X Liu X Zhou D . Bone mesenchymal stem cells are recruited via CXCL8-CXCR2 and promote EMT through TGF-β signal pathways in oral squamous carcinoma. Cell Prolif. (2020) 53:e12859. doi: 10.1111/cpr.12859 32588946 PMC7445409

[67] Drabsch Y Ten Dijke P . TGF-β signalling and its role in cancer progression and metastasis. Cancer Metastasis Rev. (2012) 31:553–68. doi: 10.1007/s10555-012-9375-7 22714591

[68] Zhang J Zheng G Zhou L Li P Yun M Shi Q . Notch signalling induces epithelial-mesenchymal transition to promote metastasis in oral squamous cell carcinoma. Int J Mol Med. (2018) 42:1909–16. doi: 10.3892/ijmm.2018.3769 30015856

[69] Pawlicka M Gumbarewicz E Błaszczak E Stepulak A . Transcription factors and markers related to epithelial–mesenchymal transition and their role in resistance to therapies in head and neck cancers. Cancers (Basel). (2024) 16:1354. doi: 10.3390/cancers16071354 38611032 PMC11010970

[70] Abdulkareem AA Shelton RM Landini G Cooper PR Milward MR . Periodontal pathogens promote epithelial-mesenchymal transition in oral squamous carcinoma cells *in vitro*. Cell Adh Migr. (2018) 12:127–37. doi: 10.1080/19336918.2017.1322253 28873015 PMC5927641

[71] Pandey P Khan F Seifeldin SA Alshaghdali K Siddiqui S Abdelwadoud ME . Targeting Wnt/β-catenin pathway by flavonoids: Implication for cancer therapeutics. Nutrients. (2023) 15:2088. doi: 10.3390/nu15092088 37432240 PMC10181252

[72] Sharma A Mir R Galande S . Epigenetic regulation of the Wnt/β-catenin signalling pathway in cancer. Front Genet. (2021) 12. doi: 10.3389/fgene.2021.681053 34552611 PMC8450413

[73] Patni AP Harishankar MK Joseph JP Sreeshma B Jayaraj R Devi A . Comprehending the crosstalk between Notch, Wnt and Hedgehog signalling pathways in oral squamous cell carcinoma - Clinical implications. Cell Oncol. (2021) 44:473–94. doi: 10.1007/s13402-021-00591-3 33704672 PMC12980738

[74] Martínez-Gascueña P Nueda ML Baladrón V . Modulation of oncogenic NOTCH signalling in highly aggressive Malignancies by targeting the γ-secretase complex: A systematic review. Cells. (2026) 15:468. doi: 10.3390/cells15050468 41827901 PMC12984106

[75] Zhu S Liu J Qiu X Cai G Gao M . TGF-β signalling in organoids: The mechanisms and applications in 3D. Cell Organoid. (2025) 1:9410015. doi: 10.26599/co.2025.9410015

[76] Katoh M Katoh M . Precision medicine for human cancers with Notch signalling dysregulation (Review). Int J Mol Med. (2020) 45:279–97. doi: 10.3892/ijmm.2019.4418 31894255 PMC6984804

[77] Deshmukh AP Vasaikar SV Tomczak K Tripathi S den Hollander P Arslan E . Identification of EMT signalling cross-talk and gene regulatory networks by single-cell RNA sequencing. PNAS. (2021) 118:e2102050118. doi: 10.1073/pnas.2102050118 33941680 PMC8126782

[78] Manfioletti G Fedele M . Epithelial–mesenchymal transition (EMT). Int J Mol Sci. (2023) 24:11386. doi: 10.3390/ijms241411386 37511145 PMC10379270

[79] Nieszporek A Skrzypek K Adamek G Majka M . Molecular mechanisms of epithelial to mesenchymal transition in tumour metastasis. Acta Biochim Pol. (2019) 66:509–20. doi: 10.18388/abp.2019_2899 31883362

[80] Wang X Song X Gao J Xu G Yan X Yang J . Hedgehog/Gli2 signalling triggers cell proliferation and metastasis via EMT and Wnt/β-catenin pathways in oral squamous cell carcinoma. Heliyon. (2024) 10:e36516. doi: 10.1016/j.heliyon.2024.e36516 39253258 PMC11382060

[81] P Thakkar J Srivastava G Shah K Arora V Behera M Gupta S . Gene expression analysis of epithelial-mesenchymal transition markers in oral submucous fibrosis fibroblasts treated with arecoline. Bioinformation. (2025) 21:3871–5. doi: 10.6026/973206300213871 41623830 PMC12859285

[82] Xie C Zhong L Feng H Wang R Shi Y Lv Y . Exosomal miR-17-5p derived from epithelial cells is involved in aberrant epithelium-fibroblast crosstalk and induces the development of oral submucosal fibrosis. Int J Oral Sci. (2024) 16:48. doi: 10.1038/s41368-024-00302-2 38897993 PMC11187069

[83] Gayathri K Malathi N Gayathri V Adtani PN Ranganathan K . Molecular pathways of oral submucous fibrosis and its progression to Malignancy. Arch Oral Biol. (2023) 148:105644. doi: 10.1016/j.archoralbio.2023.105644 36804642

[84] Zheng L Jian X Guo F Li N Jiang C Yin P . miR-203 inhibits arecoline-induced epithelial-mesenchymal transition by regulating secreted frizzled-related protein 4 and transmembrane-4 L six family member 1 in oral submucous fibrosis. Oncol Rep. (2015) 33:2753–60. doi: 10.3892/or.2015.3909 25872484

[85] Zheng L Guan ZJ Pan WT Du TF Zhai YJ Guo J . Tanshinone suppresses arecoline-induced epithelialmesenchymal transition in oral submucous fibrosis by epigenetically reactivating the p53 pathway. Oncol Res Featuring Preclinical Clin Cancer Ther. (2018) 26:483–94. doi: 10.3727/096504017X14941825760362 28550687 PMC7844836

[86] Chang Y Tsai C Lai Y Yu C Chi W Li JJ . Arecoline‐induced myofibroblast transdifferentiation from human buccal mucosal fibroblasts is mediated by <scp>ZEB</scp> 1. J Cell Mol Med. (2014) 18:698–708. doi: 10.1111/jcmm.12219 24400868 PMC4000120

[87] Lee YH Liao YW Lu MY Hsieh PL Yu CC . LINC00084/miR-204/ZEB1 axis mediates myofibroblastic differentiation activity in fibrotic buccal mucosa fibroblasts: Therapeutic target for oral submucous fibrosis. J Pers Med. (2021) 11:707. doi: 10.3390/jpm11080707 34442351 PMC8398589

[88] Dai Y Wang C Xiao Y Tan Y Ye Y Liu Y . Jiawei Danxuan Koukang and its key component quercetin intervened in OSF carcinogenesis by inhibiting the AR/eIF5A2 signalling pathway-mediated EMT. Arch Oral Biol. (2025) 173:106194. doi: 10.1016/j.archoralbio.2025.106194 39961149

[89] Mokal C Das M Hannenhalli S Agrawal P . Oral submucosal fibrosis: a comprehensive review on pathogenesis, diagnosis, therapeutics and computational advances. Front Cell Dev Biol. (2026) 13. doi: 10.3389/fcell.2025.1754209 41660015 PMC12872905

[90] Li M Deng Z Xie C Chen J Yuan Z Rahhal O . Fibroblast activating protein promotes the proliferation, migration, and activation of fibroblasts in oral submucous fibrosis. Oral Dis. (2024) 30:1252–63. doi: 10.1111/odi.14602 37357365

[91] Li C Dong X Li B . Tumour microenvironment in oral squamous cell carcinoma. Front Immunol. (2024) 15:1485174. doi: 10.3389/fimmu.2024.1485174 39744628 PMC11688467

[92] Suarez‐Carmona M Lesage J Cataldo D Gilles C . EMT and inflammation: inseparable actors of cancer progression. Mol Oncol. (2017) 11:805–23. doi: 10.1002/1878-0261.12095 28599100 PMC5496491

[93] Kondoh N Mizuno-Kamiya M . The role of immune modulatory cytokines in the tumour microenvironments of head and neck squamous cell carcinomas. Cancers (Basel). (2022) 14:2884. doi: 10.3390/cancers14122884 35740551 PMC9221278

[94] Jain SM Deka D Das A Paul S Pathak S Banerjee A . Role of interleukins in inflammation-mediated tumour immune microenvironment modulation in colorectal cancer pathogenesis. Dig Dis Sci. (2023) 68:3220–36. doi: 10.1007/s10620-023-07972-8 37277647

[95] Wieder R . Fibroblasts as turned agents in cancer progression. Cancers (Basel). (2023) 15:2014. doi: 10.3390/cancers15072014 37046676 PMC10093070

[96] Usman S Waseem NH Nguyen TKN Mohsin S Jamal A Teh MT . Vimentin is at the heart of epithelial mesenchymal transition (EMT) mediated metastasis. Cancers (Basel). (2021) 13:4985. doi: 10.3390/cancers13194985 34638469 PMC8507690

[97] Dong X Dong C Li B . Effects of macrophages in OSCC progression. Front Immunol. (2025) 15. doi: 10.3389/fimmu.2024.1517886 39877372 PMC11772471

[98] Shang GS Liu L Qin YW . IL-6 and TNF-α promote metastasis of lung cancer by inducing epithelial-mesenchymal transition. Oncol Lett. (2017) 13:4657–60. doi: 10.3892/ol.2017.6048 28599466 PMC5452994

[99] Saitoh M . Epithelial–mesenchymal transition by synergy between transforming growth factor-β and growth factors in cancer progression. Diagnostics. (2022) 12:2127. doi: 10.3390/diagnostics12092127 36140527 PMC9497767

[100] Wang H Yuan S Wang H . Current status of research on losartan in tumour therapy. J Cell Mol Med. (2026) 30:e70985. doi: 10.1111/jcmm.70985 41486497 PMC12765822

[101] Ryu D Lee JH Kwak MK . NRF2 level is negatively correlated with TGF-β1-induced lung cancer motility and migration via NOX4-ROS signalling. Arch Pharm Res. (2020) 43:1297–310. doi: 10.1007/s12272-020-01298-z 33242180

[102] Fasano M Pirozzi M Miceli CC Cocule M Caraglia M Boccellino M . TGF-β modulated pathways in colorectal cancer: new potential therapeutic opportunities. Int J Mol Sci. (2024) 25:7400. doi: 10.3390/ijms25137400 39000507 PMC11242595

[103] Takemoto A Okitaka M Takagi S Takami M Sato S Nishio M . A critical role of platelet TGF-β release in podoplanin-mediated tumour invasion and metastasis. Sci Rep. (2017) 7:42186. doi: 10.1038/srep42186 28176852 PMC5297242

[104] Wu W Chen F Cui X Yang L Chen J Zhao J . LncRNA NKILA suppresses TGF‐β‐induced epithelial–mesenchymal transition by blocking NF‐κB signalling in breast cancer. Int J Cancer. (2018) 143:2213–24. doi: 10.1002/ijc.31605 29761481

[105] Chaffer CL San Juan BP Lim E Weinberg RA . EMT, cell plasticity and metastasis. Cancer Metastasis Rev. (2016) 35:645–54. doi: 10.1007/s10555-016-9648-7 27878502

[106] Xiao L Li X Cao P Fei W Zhou H Tang N . Interleukin-6 mediated inflammasome activation promotes oral squamous cell carcinoma progression via JAK2/STAT3/Sox4/NLRP3 signalling pathway. J Exp Clin Cancer Res. (2022) 41:166. doi: 10.1186/s13046-022-02376-4 35513871 PMC9069786

[107] Hu Z Sui Q Jin X Shan G Huang Y Yi Y . IL6-STAT3-C/EBPβ-IL6 positive feedback loop in tumour-associated macrophages promotes the EMT and metastasis of lung adenocarcinoma. J Exp Clin Cancer Res. (2024) 43:63. doi: 10.1186/s13046-024-02989-x 38424624 PMC10903044

[108] Joseph JP Harishankar MK Pillai AA Devi A . Hypoxia induced EMT: a review on the mechanism of tumour progression and metastasis in OSCC. Oral Oncol. (2018) 80:23–32. doi: 10.1016/j.oraloncology.2018.03.004 29706185

[109] Tobe-Nishimoto A Morita Y Nishimura J Kitahira Y Takayama S Kishimoto S . Tumour microenvironment dynamics in oral cancer: unveiling the role of inflammatory cytokines in a syngeneic mouse model. Clin Exp Metastasis. (2024) 41:891–908. doi: 10.1007/s10585-024-10306-1 39126553 PMC11607012

[110] You Y Tian Z Du Z Wu K Xu G Dai M . M1-like tumour-associated macrophages cascade a mesenchymal/stem-like phenotype of oral squamous cell carcinoma via the IL6/Stat3/THBS1 feedback loop. J Exp Clin Cancer Res. (2022) 41:10. doi: 10.1186/s13046-021-02222-z 34991668 PMC8734049

[111] Zhong C Wang W Yao Y Lian S Xie X Xu J . TGF-β secreted by cancer cells-platelets interaction activates cancer metastasis potential by inducing metabolic reprogramming and bioenergetic adaptation. J Cancer. (2025) 16:1310–23. doi: 10.7150/jca.103757 39895802 PMC11786022

[112] de Morais EF de Oliveira LQR Marques CE Téo FH Rocha GV Rodini CO . Generation and characterization of cisplatin-resistant oral squamous cell carcinoma cells displaying an epithelial–mesenchymal transition signature. Cells. (2025) 14:1311. doi: 10.3390/cells14171311 40940722 PMC12427644

[113] Jiang X Liu B Nie Z Duan L Xiong Q Jin Z . The role of m6A modification in the biological functions and diseases. Signal Transduct Target Ther. (2021) 6:74. doi: 10.1038/s41392-020-00450-x 33611339 PMC7897327

[114] Lu HH Kao SY Liu TY Liu ST Huang WP Chang KW . Areca nut extract induced oxidative stress and upregulated hypoxia inducing factor leading to autophagy in oral cancer cells. Autophagy. (2010) 6:725–37. doi: 10.4161/auto.6.6.12423 20523123

[115] Tsai YS Lee KW Huang JL Liu YS Juo SHH Kuo WR . Arecoline, a major alkaloid of areca nut, inhibits p53, represses DNA repair, and triggers DNA damage response in human epithelial cells. Toxicology. (2008) 249:230–7. doi: 10.1016/j.tox.2008.05.007 18585839

[116] Mladenov E Magin S Soni A Iliakis G . DNA double-strand break repair as determinant of cellular radiosensitivity to killing and target in radiation therapy. Front Oncol. (2013) 3. doi: 10.3389/fonc.2013.00113 23675572 PMC3650303

[117] Srinivas US Tan BWQ Vellayappan BA Jeyasekharan AD . ROS and the DNA damage response in cancer. Redox Biol. (2019) 25:101084. doi: 10.1016/j.redox.2018.101084 30612957 PMC6859528

[118] Wang Y Su C Tseng C Chen P Nguyen HDH Chan L . Metabolic reprogramming and <scp>GM</scp> ‐ <scp>CSF</scp> secretion in areca nut‐activated fibroblasts drive oral precancer progression. Oral Dis. (2026) 32:e70236. doi: 10.1111/odi.70236 41700379 PMC13590910

[119] Singh AG Chaturvedi P . Areca nut and oral cancer. Oral Dis. (2025) 31:1467–72. doi: 10.1111/odi.14943 38566541

[120] Kaloni D Diepstraten ST Strasser A Kelly GL . BCL-2 protein family: attractive targets for cancer therapy. Apoptosis. (2023) 28:20–38. doi: 10.1007/s10495-022-01780-7 36342579 PMC9950219

[121] Yu J Fu L Wu R Che L Liu G Ran Q . Immunocytes in the tumour microenvironment: recent updates and interconnections. Front Immunol. (2025) 16. doi: 10.3389/fimmu.2025.1517959 40297580 PMC12034658

[122] Ou Z Zhu L Chen X Liu T Cheng G Liu R . Hypoxia-induced senescent fibroblasts secrete IGF1 to promote cancer stemness in oesophageal squamous cell carcinoma. Cancer Res. (2025) 85:1064–81. doi: 10.1158/0008-5472.CAN-24-1185 39661488

[123] Ding X Zheng Y Wang Z Zhang W Dong Y Chen W . Expression and oncogenic properties of membranous Notch1 in oral leucoplakia and oral squamous cell carcinoma. Oncol Rep. (2018) 40:275–285. doi: 10.3892/or.2018.6335 29620248 PMC5983926

[124] Hafız AM Doğan R Gucin Z Ozer OF Yenigun A Ozturan O . Protective and therapeutic effects of pyrrolidine dithiocarbamate in a rat tongue cancer model created experimentally using 4-nitroquinoline 1-oxide. Adv Clin Exp Med. (2020) 29:1249–54. doi: 10.17219/acem/127682 33269810

[125] Janpaijit S Sukprasansap M Tencomnao T Prasansuklab A . Anti-neuroinflammatory potential of areca nut extract and its bioactive compounds in anthracene-induced BV-2 microglial cell activation. Nutrients. (2024) 16:2882. doi: 10.3390/nu16172882 39275198 PMC11397359

[126] Mehrtash H Duncan K Parascandola M David A Gritz ER Gupta PC . Defining a global research and policy agenda for betel quid and areca nut. Lancet Oncol. (2017) 18:e767–75. doi: 10.1016/S1470-2045(17)30460-6 29208442

[127] Nithiyanantham S Arumugam S Hsu HT Chung CM Lee CP Tsai MH . Arecoline N-oxide initiates oral carcinogenesis and arecoline N-oxide mercapturic acid attenuates the cancer risk. Life Sci. (2021) 271:119156. doi: 10.1016/j.lfs.2021.119156 33548289

[128] Jin K Qian C Lin J Liu B . Cyclooxygenase-2-prostaglandin E2 pathway: a key player in tumour-associated immune cells. Front Oncol. (2023) 13. doi: 10.3389/fonc.2023.1099811 36776289 PMC9911818

[129] Yang X Zhao H Li R Chen Y Xu Z Shang Z . Stromal thrombospondin 1 suppresses angiogenesis in oral submucous fibrosis. Int J Oral Sci. (2024) 16:17. doi: 10.1038/s41368-024-00286-z 38403794 PMC10894862

[130] Heo JY Do JY Lho Y Kim AY Kim SW Kang SH . TGF-β1 receptor inhibitor SB525334 attenuates the epithelial to mesenchymal transition of peritoneal mesothelial cells via the TGF-β1 signalling pathway. Biomedicines. (2021) 9:839. doi: 10.3390/biomedicines9070839 34356903 PMC8301792

[131] Takahashi K Akatsu Y Podyma-Inoue KA Matsumoto T Takahashi H Yoshimatsu Y . Targeting all transforming growth factor-β isoforms with an Fc chimeric receptor impairs tumour growth and angiogenesis of oral squamous cell cancer. J Biol Chem. (2020) 295:12559–72. doi: 10.1074/jbc.RA120.012492 32631954 PMC7476713

[132] Tokizaki S Podyma‐Inoue KA Matsumoto T Takahashi K Kobayashi M Ibi H . Inhibition of transforming growth factor‐β signals suppresses tumour formation by regulation of tumour microenvironment networks. Cancer Sci. (2024) 115:211–26. doi: 10.1111/cas.16006 37972575 PMC10823284

[133] Hsu L Lin C Pan M Chen W . Intervention of a communication between <scp>PI3K</scp> /Akt and β‐catenin by (‑)‐epigallocatechin‐3‐gallate suppresses <scp>TGF</scp> ‐β1‐promoted epithelial‐mesenchymal transition and invasive phenotype of <scp>NSCLC</scp> cells. Environ Toxicol. (2025) 40:848–59. doi: 10.1002/tox.24475 39865447

[134] Mehta CH Paliwal S Muttigi MS Seetharam RN Prasad ASB Nayak Y . Polyphenol-based targeted therapy for oral submucous fibrosis. Inflammopharmacology. (2023) 31:2349–68. doi: 10.1007/s10787-023-01212-1 37106237 PMC10518296

[135] Avila-Carrasco L Majano P Sánchez-Toméro JA Selgas R López-Cabrera M Aguilera A . Natural plants compounds as modulators of epithelial-to-mesenchymal transition. Front Pharmacol. (2019) 10. doi: 10.3389/fphar.2019.00715 31417401 PMC6682706

[136] Hughes D Prestle J Zippel N McFetridge S Szczepan M Neubauer H . Nintedanib induces mesenchymal-to-epithelial transition and reduces subretinal fibrosis through metabolic reprogramming. Int J Mol Sci. (2025) 26:7131. doi: 10.3390/ijms26157131 40806264 PMC12346381

[137] Kamal MV Damerla RR Parida P Rao M Belle VS Dikhit PS . Expression of PTGS2 along with genes regulating VEGF signalling pathway and association with high‐risk factors in locally advanced oral squamous cell carcinoma. Cancer Med. (2024) 13:e6986. doi: 10.1002/cam4.6986 38426619 PMC10905678

[138] Kamal MV Rao M Damerla RR Pai A Sharan K Palod A . A mechanistic review of methotrexate and celecoxib as a potential metronomic chemotherapy for oral squamous cell carcinoma. Cancer Invest. (2023) 41:144–54. doi: 10.1080/07357907.2022.2139840 36269850

[139] Kumar DNA Dikhit DPS Rajan DK Usman DN Shetty DPS Mehta V . Enbloc resection of primary oral cancer involving infratemporal fossa: A systematic “out to in and top to bottom” surgical approach and outcomes. J Stomatol Oral Maxillofac Surg. (2023) 124:101515. doi: 10.1016/j.jormas.2023.101515 37247781

[140] Xu Z Huang CM Shao Z Zhao XP Wang M Yan TL . Autophagy induced by areca nut extract contributes to decreasing cisplatin toxicity in oral squamous cell carcinoma cells: Roles of reactive oxygen species/AMPK signalling. Int J Mol Sci. (2017) 18:524. doi: 10.3390/ijms18030524 28257034 PMC5372540

[141] Wang M Chen S He X Yuan Y Wei X . Targeting inflammation as cancer therapy. J Hematol Oncol. (2024) 17:13. doi: 10.1186/s13045-024-01528-7 38520006 PMC10960486

[142] Odarenko KV Zenkova MA Markov AV . The nexus of inflammation-induced epithelial-mesenchymal transition and lung cancer progression: A roadmap to pentacyclic triterpenoid-based therapies. Int J Mol Sci. (2023) 24:17325. doi: 10.3390/ijms242417325 38139154 PMC10743660

[143] Kamal MV Prabhu K Sharan K Pai A Chakrabarty S Damerla RR . Investigation of the molecular mechanisms of paraoxonase‐2 mediated radiotherapy and chemotherapy resistance in oral squamous cell carcinoma. Clin Transl Sci. (2025) 18:e70201. doi: 10.1111/cts.70201 40134131 PMC11936840

[144] Wang TY Peng CY Lee SS Chou MY Yu CC Chang YC . Acquisition cancer stemness, mesenchymal transdifferentiation, and chemoresistance properties by chronic exposure of oral epithelial cells to arecoline. In: Oncotarget Albany: Impact Journals (2016). Available online at: https://www.impactjournals.com/oncotarget/ (Accessed June 6, 2026). 10.18632/oncotarget.11432 PMC5356645 27557511

